# Does pain influence control of muscle force? A systematic review and meta‐analysis

**DOI:** 10.1002/ejp.4716

**Published:** 2024-08-23

**Authors:** Michail Arvanitidis, Deborah Falla, Andy Sanderson, Eduardo Martinez‐Valdes

**Affiliations:** ^1^ Centre of Precision Rehabilitation for Spinal Pain (CPR Spine) School of Sport, Exercise and Rehabilitation Sciences, College of Life and Environmental Sciences, University of Birmingham Birmingham UK; ^2^ Department of Sport and Exercise Sciences Institute of Sport, Manchester Metropolitan University Manchester UK

## Abstract

**Background and Objective:**

In the presence of pain, whether clinical or experimentally induced, individuals commonly show impairments in the control of muscle force (commonly known as force steadiness). In this systematic review and meta‐analysis, we synthesized the available evidence on the influence of clinical and experimental pain on force steadiness.

**Databases and Data Treatment:**

MEDLINE, EMBASE, PubMed, CINAHL Plus and Web of Science databases were searched from their inception to 19 December 2023, using MeSH terms and pre‐selected keywords related to pain and force steadiness. Two independent reviewers screened studies for inclusion and assessed their methodological quality using a modified Newcastle–Ottawa risk of bias tool.

**Results:**

In total, 32 studies (19 clinical pain and 13 experimental pain) were included. Meta‐analyses revealed reduced force steadiness in the presence of clinical pain as measured by the coefficient of variation (CoV) and standard deviation (SD) of force (standardized mean difference; SMD = 0.80, 95% CI = 0.31–1.28 and SMD = 0.61, 95% CI = 0.11–1.11). These findings were supported by moderate and low strength of evidence respectively. In the presence of experimental pain, meta‐analyses revealed reductions in force steadiness when measured by the CoV of force but not by the SD of force (SMD = 0.50, 95% CI = 0.01–0.99; and SMD = 0.44, 95% CI = −0.04 to 0.92), each supported by very low strength of evidence.

**Conclusions:**

This work demonstrates that pain, particularly clinical pain, impairs force steadiness. Such impairments likely have clinical relevance and could become targets for treatment when managing people experiencing musculoskeletal pain.

**Significance Statement:**

This systematic review and meta‐analyses enhances our understanding of motor impairments observed in people experiencing musculoskeletal pain. It underscores the significance of incorporating force steadiness assessment when managing individuals experiencing musculoskeletal pain. Additionally, it suggests that future research should explore the potential benefits of force steadiness training in alleviating patients' symptoms and enhancing their functional performance. This could potentially lead to the development of innovative therapeutic approaches for individuals suffering from musculoskeletal pain.

## 1 INTRODUCTION

During voluntary submaximal movements, the force exerted by individuals inherently varies, fluctuating around an average value (Enoka et al., 2003). Such force variability stems from neuromuscular noise and other external factors (Oomen & van Dieën, 2017). The ability to generate steady force output during submaximal voluntary contractions is termed force (or torque) steadiness (Tracy & Enoka, 2002). Minimizing these force fluctuations is vital for maintaining physical functionality, and its deterioration could compromise the precision of voluntary actions, affecting joint stability, coordination and overall movement (Deering et al., 2017; Maenhout et al., 2012).

The smooth generation of force relies heavily on the perception of force, a component of proprioception (Ager et al., 2020). Proprioception is commonly compromised in the presence of acute, chronic or experimentally induced pain, likely due to altered afferent information from the affected area and/or central changes (Ager et al., 2020; Lee et al., 2010; Röijezon et al., 2015; Salahzadeh et al., 2013; Sjölander et al., 2008; Treleaven, 2008). Proprioceptive and nociceptive inputs share a complex neurological pathway and may interfere with each other at different levels of processing (Ager et al., 2020). Moreover, musculoskeletal injuries can further impair proprioception by structural damage to tissue (Röijezon et al., 2015). Therefore, pain‐induced proprioceptive alterations could potentially degrade our ability to control muscle force.

Assessment of force steadiness typically involves measuring the variability of the force output, quantified by the standard deviation (SD) or coefficient of variation (CoV) of the force or torque signal over time, which serve as indicators of absolute and relative neuromuscular control (Fiogbé et al., 2019), where higher SD and CoV values denote less force steadiness. Additionally, measures such as approximate and sample entropy are sometimes used (SampEn and ApEn), as they offer insights into the complexity and regularity of the force signal. Lower entropy values suggest a more predictable and regular force output, while higher values indicate greater complexity and potential instability in motor control (Smith et al., 2021).

The current literature offers inconsistent findings on the effect of pain, be it clinical or experimental, on force steadiness. Some studies suggest that conditions such as chronic low back pain (CLBP) (Arvanitidis et al., 2022; Miura & Sakuraba, 2014), chronic neck pain (CNP) (Muceli et al., 2011) or patellofemoral pain syndrome (PFP) (Ferreira et al., 2021) are associated with decreased force steadiness in specific isometric tasks, while others report no such changes (Camargo et al., 2009; Crowley et al., 2022; Hirata et al., 2015; Martinez‐Valdes et al., 2020). Similarly, studies have shown that experimentally induced pain is associated with reductions in isometric fifth finger abduction (Farina et al., 2012) and knee extension (Poortvliet et al., 2019) force steadiness, while others did not observe changes in dorsiflexion steadiness in the presence of experimentally induced pain (Martinez‐Valdes et al., 2021, 2020). Given this variability, the aim of this systematic review is to synthesize the available evidence to understand if pain, whether it be clinical or experimental, influences force steadiness during isometric and dynamic voluntary contractions.

## 2 METHODS

This review was conducted in line with the 2020 guidelines of the preferred reporting items for systematic reviews and meta‐analyses (PRISMA) (Page et al., 2021) as evidenced in Data S1. The protocol for this review was pre‐registered on the International Prospective Register of Systematic Reviews (PROSPERO) with the registration number CRD42020196479 on 21 July 2020 and has been published (Arvanitidis et al., 2021). While adherence to the published protocol was rigorous, minor deviations were necessary during data synthesis. Specifically, it was not possible to subgroup data based on the muscle/joint assessed or the implementation of visual feedback. To address the variability among studies, additional heterogeneity measures were evaluated. Furthermore, the standardized mean difference (SMD) was utilized for all studies instead of odds ratios, as it was considered more appropriate for the analysis.

### 2.1 Eligibility criteria

The inclusion criteria for this systematic review were defined by using a modified version of the PICOS framework, which included population, indicator, comparison, outcomes and study design (Smith et al., 2021). Due to the nature of the studies, the original term ‘Intervention’ was replaced with ‘Indicator’, in line with previous adaptations for observational studies (Devecchi et al., 2019; Devecchi et al., 2021).

#### 2.1.1 Population

Studies were eligible for inclusion if they focused on adults (≥18 years) with musculoskeletal pain (clinical), defined as *‘pain experienced in muscles, tendons, bones or joints that arises from an underlying disease classified elsewhere’* – chronic secondary musculoskeletal pain (Perrot et al., 2019) or *‘pain that is characterised by significant emotional distress or functional disability, and cannot be attributed directly to a known disease or damage process’* – chronic primary musculoskeletal pain (Nicholas et al., 2019; Perrot et al., 2019). Additionally, studies investigating experimentally induced musculoskeletal pain, that is, pain arising from the sensitization of nociceptors in subcutaneous tissues through electrical or chemical stimulation of the muscle and/or joint (Reddy et al., 2012), were also included. These models were specifically chosen because they can induce deep tissue pain, closely mirroring the characteristics of clinical musculoskeletal pain (Graven‐Nielsen et al., 1997). A requirement for inclusion was a control group of asymptomatic individuals or a crossover design where the same participants served as their controls in a pain‐free state. There were no restrictions based on gender or ethnicity. The exclusion criteria applied to studies involving pain from exercise‐induced soreness (delayed onset of muscle soreness; DOMS) or fatigue, or thermal models, as well as participants with neuromuscular and neurological disorders, systemic inflammatory diseases or a history of surgery/fracture in the area of interest, to eliminate confounding factors.

#### 2.1.2 Indicator

Studies selected included those using dynamometers or similar instruments, such as force or torque sensors, to assess steadiness of force or torque or related measures of variability. Studies employing force platforms for assessing postural steadiness, such as during standing, were not considered. Inclusion was not limited by the provision of visual feedback during trials; studies with and without visual feedback on the force or torque output were eligible for inclusion. All forms of contractions, whether measured at an absolute level or relative to the individual's maximal voluntary contraction (MVC), were considered and there was no restriction for the side of the body assessed (i.e. dominant or non‐dominant).

#### 2.1.3 Comparison

Studies that were selected included those that compared force or torque steadiness in voluntary contractions between painful and non‐painful states. Comparisons were conducted either within participants, as in studies of experimentally induced pain, or between groups, such as between pain‐affected participants and a control group. Additionally, comparisons encompassed multiple assessments and measurements taken both pre‐ and post‐task.

#### 2.1.4 Outcomes

The primary outcome assessed was the measurement of force or torque steadiness. For this purpose, the following force steadiness measures were considered: the coefficient of variation (CoV) and SD of force or torque, as well as the root mean square (RMS) of the force or torque signal during force template matching tasks. Studies that evaluated torque complexity through measures such as SampEn or ApEn were also included. The inclusion was restricted to quantitative studies that measured force or torque steadiness or variability. Studies that investigated other aspects of muscle force control, such as force accuracy (i.e. the ability of an individual to produce a force that matches a specified target force as closely as possible; force reproduction tasks, etc.) (Pranata et al., 2017), were excluded.

#### 2.1.5 Study design

**FIGURE 1:**
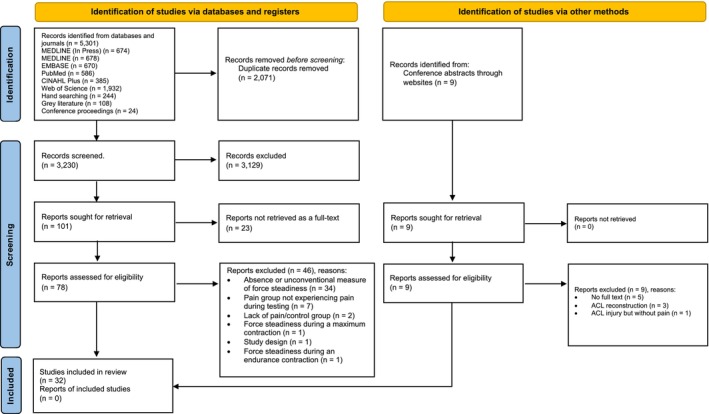
PRISMA flow diagram, adapted from Page et al. (2021). PRISMA, preferred reporting items for systematic reviews and meta‐analyses.

Scoping searches indicated that observational studies primarily explore the research question of this systematic review. Consequently, only observational studies employing quantitative methods, specifically cross‐sectional, case–control and cohort studies, were included. Non‐original works such as systematic and narrative reviews, as well as other study types, were excluded. To reduce potential bias, the search encompassed studies in all languages. However, due to constraints of time and resources, studies not written in English were excluded. Details of any excluded studies are documented in the PRISMA flow diagram (Figure 1).

### 2.2 Information sources

Electronic databases were searched from their inception to 19 December 2023 and included MEDLINE (Ovid Interface), EMBASE (Ovid Interface), PubMed, CINAHL Plus (EBSCO Interface) and Web of Science (Clarivate Analytics). While the ZETOC database was initially considered for the search, it was ultimately not accessed as the database was closed as of 1 August 2022. Tailored search strategies were formulated for each database, incorporating Medical Subject Headings (MeSH) where applicable to refine and optimize the search (Richter & Austin, 2012).

In parallel with the electronic database search, hand searches of select journals were also performed. These journals encompassed PAIN, Journal of Physiology, Journal of Neurophysiology, Acta Physiologica, Journal of Electromyography and Kinesiology, Clinical Biomechanics, Muscle & Nerve, Journal of Orthopaedic & Sports Physical Therapy, Journal of Science and Medicine in Sport, Isokinetics and Exercise Science and Journal of Applied Physiology. Efforts were made to identify any unpublished but relevant studies by directly contacting subject matter experts in the field. To mitigate the risk of publication bias, grey literature was also reviewed, accessed through the British National Bibliography for Report Literature, OpenGrey database, ProQuest Dissertations & Theses Global and EThOS.

Additionally, proceedings from the World Congress of Biomechanics, the International Society of Biomechanics, the International Society of Electrophysiology and Kinesiology and the World Confederation for Physical Therapy were scrutinized, covering the years 2016 to 2023. Authors of studies that appeared to be eligible were contacted to confirm the publication status of their work. To further reduce the chance of publication bias, the reference lists of all included studies were manually reviewed to identify any relevant studies that might have been missed during the initial search.

### 2.3 Search strategy

The lead author (MA) conducted the search without imposing limitations on date, format, design, geographical area or language. To guarantee both comprehensiveness and reproducibility of the search, it was crafted following initial scoping searches and with guidance from a skilled Health Sciences Librarian, a member of the University of Birmingham's Research Skills Team. The full electronic search approach for the MEDLINE (Ovid Interface) database is detailed in Data S2. This approach combined MeSH terms and keyword searches to optimize the search yield. The search was slightly adapted for different databases, but consistency was ensured. These changes involved alterations in MeSH terms and syntax. For example, the ‘ADJ’ operator was switched to ‘NEAR’ in the Web of Science database and to ‘N’ in the CINAHL Plus (EBSCO Interface) database. The search strategies for all databases are detailed in Data S2.

### 2.4 Selection process

All search results were imported into EndNote Version 20 (Clarivate Analytics) by one reviewer (MA) for data management. Duplicates were automatically removed by the software. For screening, the search results were also made available in separate folders for each independent reviewer (MA and AS) and were assessed using a pre‐tested screening form. Titles and abstracts were initially screened by two independent reviewers (MA and AS) to categorize studies as definitely eligible, ineligible or doubtful. Any disagreements or doubtful studies were discussed between reviewers, and in cases of disagreement or uncertainty, a third reviewer (EM‐V) was consulted.

The two reviewers independently conducted both stages of the selection process, including the screening of titles/abstracts and full‐text evaluation. Any disagreement between reviewers was resolved by the third reviewer (EM‐V). The level of agreement between reviewers was evaluated using the kappa (*κ*) statistic.

### 2.5 Data collection process and data items

Data extraction was conducted by one reviewer (MA) using a tested extraction form, designed to gather essential elements aligned with the review's objectives. The accuracy of the extracted data was verified by a second reviewer (AS). When additional clarification was needed for ambiguous or incomplete data, authors were contacted with a 2‐week reply window. Failure to respond led to the study's exclusion for ambiguity.

As detailed earlier, data relevant to each element of our inclusion criteria were extracted. When studies included additional groups not relevant to our review, only data relevant to our specific interest groups were extracted.

### 2.6 Risk of bias assessment

Two independent reviewers (MA and AS) employed two adapted versions of the Newcastle–Ottawa Scale (NOS) to assess risk of bias; one version for clinical pain studies adapted from the case–control version and another for experimental pain studies adapted from the cohort version. It is important to note that the experimental pain studies had repeated measures or cross‐over design and were experimental in nature. We selected to use the adapted cohort version of the NOS for these studies to ensure consistency within the manuscript, to adhere to our previously published protocol (Arvanitidis et al., 2021) and because it was considered to be more appropriate to the case–control version. The changes and justifications for each are included in Data S3. Briefly, in both versions of the tool, the *‘exposure’* domain in the original NOS was specifically modified to an *‘outcome’* domain to fit the nature of our studies better while maintaining the essence of the questions. Scoring ranged from 0 to 9, with higher scores indicating lower risk of bias. Quality categorization for each study was then done as *‘good’*, *‘fair’* or *‘poor’*, according to established thresholds. Further information on our rationale for selecting the NOS tool for the risk of bias can be found in our published protocol (Arvanitidis et al., 2021).

### 2.7 Synthesis methods

Both narrative and meta‐analyses methods were used to synthesize the data from the included studies, to explore the influence of pain on force steadiness (Deeks et al., 2019; McKenzie et al., 2019). Studies were grouped by the type of pain (clinical or experimental) for methodological consistency, and the narrative synthesis included the outline and tabulation of study characteristics (McKenzie et al., 2019). This allowed studies, not included in the meta‐analysis, to be interpreted and the influence of their findings to be considered, alongside other clinical‐ or methodological‐related information from each study.

In preparation for the meta‐analysis, studies were further subgrouped based on the outcome investigated (e.g. CoV or SD of force/torque). Considering our research question and the limited number of studies per muscle/joint, subgroup analysis by muscle/body region or type of contraction was not performed. Only studies with available or retrievable mean ± SD data on force steadiness were included for meta‐analysis. Before conducting the meta‐analysis, descriptive statistics were used to appropriately format the data. For example, if a study reported the standard error (SE) instead of the SD, this was calculated by using the following formula.

$$\mathrm{SD}=\mathrm{SE}\times \sqrt{N}$$

Additionally, for within‐study calculations where multiple observations per group were reported, a weighted average was employed to adjust for any differences in sample size across these observations. A pooled SD was used to pool the individual variances of these observations into a cohesive measure of dispersion. The formulas used are reported below:

$${\bar{x}}_{w}=\frac{\sum_{i=1}^{k}\left(x_{i}\cdot n_{i}\right)}{\sum_{i=1}^{k}n_{i}},{\mathrm{SD}}_{\text{pooled}}=\sqrt{\frac{\sum_{i=1}^{k}\left(\left(n_{i}-1\right)\cdot SD_{i}^{2}\right)}{\sum_{i=1}^{k}\left(n_{i}-1\right)}}$$

where for the formula for the weighted mean (${\bar{x}}_{w}$), *x*_*i*_ is the mean of observation *i* and *n*_*i*_ is the sample size associated with observation *i* and *k* the total number of observations. For the SD pooled formula (${\mathrm{SD}}_{\text{pooled}}$), *SDi* is the standard deviation of observation *i*, *n*_*i*_ is the sample size for observation *i* and *k* is the number of observations. All meta‐analysis procedures and forest plot generation were performed using the R software (4.2.1) (Team, 2022) and the ‘*meta*’ package (Balduzzi et al., 2019) by selecting a multi‐level random‐effects model to adequately accommodate the anticipated heterogeneity across the included studies and adjust the weight of studies that were included more than one time in the model (Deeks et al., 2019). Data pooling was performed using the SMD and the *‘metacont’* function within the ‘*meta*’ package which is designed for continuous outcomes using 95% confidence intervals in line with others (Devecchi et al., 2021; Pethick et al., 2022) and guidelines (Deeks et al., 2019). The Knapp–Hartung adjustment (Knapp & Hartung, 2003) was also used to calculate the 95% CI around the pooled effect estimate (Jackson et al., 2017).

Even though we implemented a random‐effects model to address heterogeneity statistically, it is important to recognize that this approach does not eliminate heterogeneity; it merely accounts for it (Deeks et al., 2019). Therefore, as recommended (Deeks et al., 2019; IntHout et al., 2016) we further explored heterogeneity within the data, by calculating and reporting additional measures. Rather than relying solely on the *I* ^*2*^ statistic, which has some limitations in providing insights into the nature of heterogeneity, we also incorporated *τ* ^2^ (*τ* ^2^). *τ* ^2^, which is a quantitative estimate of the extent to which the true effects vary across studies (Deeks et al., 2019). As recommended (Deeks et al., 2019; IntHout et al., 2016; Jackson et al., 2017; Teichert et al., 2023), the between‐study variance (*τ* ^2^) was estimated using the restricted maximum‐likelihood estimator. In accordance with recent recommendations (IntHout et al., 2016), we also incorporated the calculation of the prediction interval for the pooled effect size into our analysis. This approach offers a broader perspective on the potential range of true effects within comparable studies (Deeks et al., 2019; IntHout et al., 2016). By including the prediction interval alongside the summary estimate and confidence interval (CI), our reporting illustrates the expected range of true effects in future studies and can simplify its clinical interpretation (Deeks et al., 2019; IntHout et al., 2016).

### 2.8 Sensitivity analyses

Due to the expected high heterogeneity commonly seen in pain studies, we also conducted a sensitivity analysis (Deeks et al., 2019). This involved excluding each study in turn to check the consistency of our meta‐analysis results, as detailed in Data S4. This step confirmed the findings' robustness and assessed the impact of excluding each study on overall heterogeneity.

Additionally, considering that the presence of outliers and influential cases may affect the validity and robustness of the conclusions from a meta‐analysis, we quantitatively explored potential outliers using deletion diagnostics known from linear regression (i.e. externally standardized residuals, DFFITS values, Cook's distances, covariance ratios, DFBETAS values, estimates of *τ* ^2^ and Q when each study is removed in turn, diagonal elements of the hat matrix and the weights (%) given to the observed outcomes during model fitting) that can also be adapted to the context of meta‐analysis as described previously (Viechtbauer, 2010; Viechtbauer & Cheung, 2010). This was performed using the *‘influence’* function from the *‘metafor’* package in R (Viechtbauer, 2010). Sensitivity analyses were also performed by removing studies that did not use visual feedback during the contractions to maintain consistency, as the presence or absence of visual feedback could have been a confounding factor. This approach allowed us to standardize the analysis across the more commonly reported condition of ‘with visual feedback’. It was not possible to perform a sensitivity analysis excluding studies with visual feedback due to the limited number of studies assessing force steadiness without visual feedback. Additionally, we removed studies of poor quality to assess their influence on the overall results. Using this comprehensive approach to identify influential cases and conduct sensitivity analyses, we aimed to ensure the robustness of our meta‐analysis results.

### 2.9 Certainty of evidence

The cumulative evidence from the meta‐analyses (for each outcome and type of pain) was also appraised by the two independent reviewers (MA and AS) using the grading of recommendations assessment, development and evaluation (GRADE) method, assessing cumulative strength and quality. This process involved five steps, as described previously (Goldet & Howick, 2013), with the final evidence quality classified as *‘high’*, *‘moderate’*, *‘low’* or *‘very low’*. Observational studies initially received a low‐quality rating, which was then adjusted based on certain criteria. Evidence was upgraded for substantial effect sizes and clear dose–response trends. Conversely, risk of bias, study inconsistencies, imprecision, indirectness and publication bias led to downgrades (Balshem et al., 2011). Publication bias was assessed by generating funnel plots and visually inspecting their symmetry, as well as using Egger's regression test to quantify the presence of publication bias (Egger et al., 1997; Sterne & Egger, 2001). We applied this quality assessment across all studies and subgroups in our review, leading to tailored evidence interpretation recommendations in line with guidelines for observational studies (Dekkers et al., 2019; Mueller et al., 2018).

## 3 RESULTS

### 3.1 Search results and study selection

The results of the search and selection process are outlined in Figure 1. The database searches initially resulted in 5301 records, with nine additional records/abstracts identified through targeted handsearching of conferences. After removing duplicates, 3230 records from databases and 9 from conference hand searching underwent title and abstract screening, with the inter‐reviewer agreement reflected by a high kappa coefficient *κ* = 0.9. Full‐text screening was then applied to 78 records from the databases/journal search, excluding the other 9 records for reasons specified in Figure 1. The inter‐reviewer agreement for the full‐text screening was perfect, with *κ* = 1.00. The review ultimately included 32 studies.

Some studies that may seem relevant for this review were excluded. Specifically, seven studies (Hollman et al., 2021; Maenhout et al., 2012; Ross et al., 2015; Saccol et al., 2014; Sarah Ward & Bryant, 2018; Zanca et al., 2010; Zanca et al., 2013) were excluded because the patient groups did not experience or report pain during the assessment. Moreover, one study (Dinsdale et al., 2023) was excluded because the assessment of force steadiness was performed during an endurance contraction, and muscle fatigue could have been a confounding factor. Lastly, another study was excluded due to its randomized controlled trial design, which was outside the methodological scope of this review (Mista et al., 2016). Furthermore, this study was quite different from the others included, as it consisted of two groups that attended three experimental sessions (day 0, 2 and 4), with one group receiving nerve growth factor and the other isotonic saline. The only usable data would have been from day 2, but even the control group (i.e. the individuals who received the isotonic saline injection) experienced some minimal pain, and a learning effect could not be excluded, considering that the individuals had already performed the task twice on day 0.

### 3.2 Characteristics of included studies

This review included 32 studies (19 clinical pain and 13 experimental pain), and their characteristics are summarized in Tables 1 and 2. Most studies used CoV and/or SD of force to quantify force steadiness, while only a few used other measures such as SampEN and ApEn for force complexity and root mean square (RMS) for force steadiness.

**TABLE 1:** Characteristics of studies examining whether people with clinical pain show differences in force steadiness with respect to asymptomatic controls.

Study	Patient population characteristics & self‐reported measures	Control population characteristics & self‐reported measures	Force steadiness task and contraction intensity	Outcome measure	Results
Arvanitidis et al. (2022)	**15 CLBP participants** (7 M, 8 F, 27.1 ± 9.3 years, 69.3 ± 10.1 kg, 169.9 ± 5.7 cm); **average pain intensity (NRS**: **0–10)**: 2.5 ± 2.2; **ODI (%):** 14.9 ± 7.5; **SF‐36 (general health score):** 64.1 ± 19.6; **TSK:** 34.7 ± 5.2	**15 asymptomatic controls** (8 M, 7 F, 27.4 ± 4.9 years, 73.7 ± 10.4 kg, 171.3 ± 5.4 cm); **average pain intensity (NRS**: **0–10):** 0.0 ± 0.0; **ODI (%):** 0.0 ± 0.0; **SF‐36 (general health score):** 89.6 ± 6.8; **TSK:** 28.1 ± 6.0	Isometric trunk extension contractions (90°) at **20% MVC** and **50% MVC**, with visual feedback provided by a monitor placed 1.5 m in front of the participants. Participants were seated on an isokinetic dynamometer (Biodex System 3 Pro).	**SD (%), CoV (%)**	↓ Torque steadiness in people with CLBP (CoV and SD of torque, main effect of group: *F* = 5.327, *p* = 0.029, *ηp* ^2^ = 0.160 and *F* = 4.368, *p* = 0.046, *ηp* ^2^ = 0.135 respectively).
Arvanitidis et al. (2024)	**20 CLBP participants** (10 M, 10 F, 31.1 ± 6.9 years, 75.3 ± 18.4 kg, 170.6 ± 9.9 cm); **average pain intensity (NRS**: **0–10):** 3.8 ± 1.6; **ODI (%):** 21.5 ± 13.4; **SF‐36 (general health score):** 58.0 ± 19.2; **TSK:** 36.6 ± 7.4	**20 asymptomatic controls** (10 M, 10 F, 28.6 ± 3.9 years, 70.6 ± 13.4 kg, 171.9 ± 7.4 cm); **average pain intensity (NRS**: **0–10):** 0.0 ± 0.0; **ODI (%):** 0.0 ± 0.0; **SF‐36 (general health score):** 81.5 ± 9.8; **TSK:** 24.8 ± 4.4	Concentric and eccentric trunk flexion and extension contractions on an isokinetic dynamometer (Biodex System 3 Pro). Total range of motion: 50°. Four contractions at **25%MVC** and three at **50%MVC**, with visual feedback provided by a monitor placed 1.5 m in front of the participants.	**SD (%), CoV (%)**	↓ **Trunk extension** torque steadiness in people with CLBP (CoV and SD of torque, condition effect: MD = 2.05%, *F* = 40.01; MD = 0.60%, *F* = 25.31, respectively; *p* < 0.0001 for both). This was evident during both the eccentric and concentric contractions and at both low and high torque levels. ↓ **Trunk flexion** torque steadiness in people with CLBP (CoV and SD of torque, condition effect, *F* = 41.11, MD = 3.36%; *F* = 53.31, MD = 1.31%, respectively; *p* < 0.0001 for both). This was evident during both the eccentric and concentric contractions and at both low and high torque levels.
Bandholm et al. (2006)	**Nine participants with SIS** (28.2 ± 5.3 years; range, 21–38 years; physically active despite shoulder pain); **mean duration of symptoms:** 27.6 ± 27.7 (range: 5–72 months); **pain intensity during contractions (VAS**: **0–10): ISO TF20:** 1.8 ± 1.3, **ISO TF27.5:** 2.1 ± 1.4, **ISO TF35:** 2.4 ± 2.1; **DYN TF20:** 2.5 ± 1.2, **DYN TF27.5:** 2.9 ± 2.0, **DYN TF35:** 3.6 ± 2.3.	**Nine asymptomatic controls** (27.7 ± 4.2 years; range, 22–37 years)	Isometric (90°), isokinetic (dynamic, 30–120° at 15°/s) shoulder abduction contractions on an isokinetic dynamometer (Kin‐Com) with an oscilloscope placed 1 m away from their seat. Seated, with dominant arm at 90° elbow flexion, neutral lower arm. Target forces: **20, 27.5, 35% MVC**.	**SD (N), CoV (%)**	↓ Reduced force steadiness in people with SIS at 35% MVC during concentric contractions for both SD and CoV of force (*p* < 0.05 and *p* = 0.03). No differences during isometric contractions.
Camargo et al. (2009)	**27 participants with unilateral SIS** (33.48 ± 9.94 years; 9 F, 18 M); dominant involved side (*N* = 17), non‐dominant involved side (*N* = 10); **duration** of shoulder pain (self‐reported): 31.31 ± 33.09 months; **DASH questionnaires**: **SIS (dominant side):** 22.64 ± 17.53 (1.66–50.83); **SIS (non‐dominant involved):** 18.00 ± 12.47 (6.66–44.16)	**23 asymptomatic controls** (32.26 ± 9.04 years; 8 F, 15 M); **DASH questionnaire:** 0.98 ± 1.79 (0.00–5.83)	Isometric shoulder abduction (80°) on an isokinetic dynamometer from a seated position. Torque steadiness was assessed at **35% MVC**. Visual feedback was provided via a monitor. The SIS group was divided into two groups: (1) SIS with the dominant involved side and (2) the non‐dominant involved side	**SD (N), CoV (%)**	No changes in shoulder abduction torque steadiness in individuals with SIS (SD, CoV; *p* > 0.05).
Chen et al. (2023)	**15 people with CLE** (10 M, 5 F, 46.5 ± 6.3 years, 75.7 ± 13.1 kg, 168.3 ± 8.2 cm); **pain intensity (VAS**: **0–10)**: 7.1 ± 1.8; **DASH score:** 35.1 ± 16.1.	**15 asymptomatic controls** (10 M, 5 F, 45.3 ± 2.5 years, 75.1 ± 14.9 kg, 169.1 ± 6.1 cm); **pain intensity (VAS**: **0–10):** N/A; **DASH score**: N/A.	Isometric wrist extension against a force sensor (model: MB‐100, Interface Inc., Scottsdale, AZ, United States). Visual feedback was provided by a monitor. Target force: up to **75%MVC** (8 s ramp‐up and 8 s ramp‐down). Each participant performed three trials.	**SampEn, RMS**	↓ Reduced force steadiness (RMS, ramp‐up phase), in people with CLE RMS (*p* = 0.001). No differences when measured as SampEn during the ramp‐up phase (*p* = 0.226).
Crowley et al. (2022)	**34 men with IAT** (age 43.7 ± 10.02 years, weight 89.6 ± 16.3 kg); **VISA‐A (0–100):** 54.1 ± 16.6; **Pain Catastrophizing Scale:** 12.6 ± 11.2; **TSK:** 36.4 ± 6.6; **Single leg calf raise pain (NRS):** 2.8 ± 2.3	**34 healthy men** (age 42.8 ± 8.9 years, weight 87.2 ± 9.7 kg); **VISA‐A (0–100):** 97.7 ± 0.5; **Pain Catastrophizing Scale:** 5.2 ± 6.8; **TSK:** 29.7 ± 7.2	Isometric plantar flexion (0° dorsiflexion; knee flexed to 50°) on a custom‐designed chair with foot plates. Isometric plantar flexion contractions at **10% MVC.** Real‐time force output displayed on a monitor.	**CoV (%)**	No between‐group differences were found for plantar flexor force steadiness (*p* = 0.09, *d* = 0.44).
Falla et al. (2010)	**Nine women** (age, mean ± SD: 40.4 ± 3.5 years; height: 170.8 ± 5.5 cm; weight: 73.7 ± 10.1 kg) with **chronic, non‐traumatic neck pain** greater than 3 months (years, mean ± SD: 12.3 ± 11.1; range: 1–33); **NDI (0–50):** 16.5 ± 8.8 (range: 5–31); **average pain intensity rated on VAS (0–10):** 4.3 ± 1.5 (range: 2.0–6.9).	**Nine women** were recruited as **controls** (age, mean ± SD: 35.4 ± 7.5 years; height: 164.8 ± 7.7 cm; weight: 65.0 ± 12.3 kg).	Isometric neck contractions (seated) in 8 different angles and circular neck contractions (45° intervals) 0‐360° with a load of **15 and 30 N**. Circular contractions in the horizontal plane at **15 and 30 N** force, from 0 to 360° were also performed (clockwise and counterclockwise). Real‐time visual feedback was provided via an oscilloscope.	**CoV (%)**	↓ Reduced force steadiness in people with neck pain (CoV) during brief constant force contractions and circular contractions performed at 30 N (*p* = 0.03; SNK: *p* = 0.002).
Ferreira et al. (2021)	**30 people with PFP**: **Age (years):** 21.26 ± 3.06; **body mass (kg):** 62.66 ± 10.61; **height (m):** 1.62 ± 0.05; **BMI**: 23.80 ± 3.86; **worst pain level in the last month, VAS (0–100):** 51.67 ± 16.15; **pain level during knee extension – VAS (0–100):** 28.00 ± 19.93; **pain level during hip abduction, VAS (0–100):** 23.66 ± 26.45; **symptoms duration (months):** 60.53 ± 54.01	**30 asymptomatic controls**: **Age (years):** 22.10 ± 2.78, **body mass (kg):** 57.66 ± 8.76, **Height (m):** 1.61 ± 0.06, **BMI:** 22.15 ± 3.07	Knee extensor and hip abductor force steadiness on an isokinetic dynamometer (Biodex System 4 Pro). **Knee extension:** seated, with the hip and untested knee stabilized at 90° flexion. Knee joint angle of 60° flexion during testing. **Hip abduction:** side lying, with untested hip and knee flexed and fixed. Hip abductor testing was done at 20° hip abduction. Submaximal isometric force target set at **10% MVC.** Real‐time visual feedback was provided via a monitor.	**CoV (%)**	↓ Reduced knee extensor and hip abductor isometric force steadiness in women with PFP (Cohen's *d* = 2.04; 95% CI = [5.25–8.82] and Cohen's *d* = 2.35; 95% CI [10.98–17.18], respectively).
Hortobágyi et al. (2004)	**20 participants with OA** (***N*** = 15 F, 5 M), **age (years):** 57.5 ± 7.3 (43–68), **mass (kg):** 86.8 ± 18.3 (62–110), **height (m):** 1.64 ± 0.09 (1.50–1.82), **BMI (kg/m** ^**2**^ **):** 29.3 ± 3.2 (24–35); level of pain for the week before testing (mean ± SD: 1.87 ± 0.32), immediately before (1.96 ± 0.38) and immediately after (1.86 ± 0.29) the testing session (0–4 scale, where 0 = no pain, 4 = excruciatingly painful). **Level walking:** 17.6 ± 2.1 s; **stair descent:** 10.2 ± 2.3 s; **stair ascent:** 12.8 ± 3.2 s; **get up and go:** 9.3 ± 1.1 s; **mean:** 12.5 ± 2.2 s	**20 asymptomatic controls** (***N*** = 15 F, 5 M), **age (years):** 56.8 ± 5.0 (45–66), **mass (kg):** 81.2 ± 12.5 (60–96), **height (m):** 1.67 ± 0.07 (1.52–1.83), **BMI (kg/m** ^**2**^ **):** 28.3 ± 3.0 (23–33) No knee pain. **Level walking**: 11.5 ± 1.1 s; **stair descent:** 5.8 ± 0.9 s; **stair ascent:** 7.2 ± 0.8 s; **get up and go:** 5.7 ± 0.7 s; **mean:** 7.6 ± 0.9 s	Knee extension steadiness tasks on an isokinetic dynamometer (KinCom). Three types of contractions: eccentric, concentric and isometric. In seated position, the dominant leg was tested. Steadiness tasks using target forces of 50 and 100 N. During testing, real‐time visual feedback for the force output was provided to all participants on a computer monitor.	**SD (N)**	↓ Quadriceps force steadiness in people with knee OA during dynamic contractions. OA patients were unsteady at both 50‐N and 100‐N target forces. No reductions were observed during isometric contractions.
Magni et al. (2021)	**62 participants with symptomatic hand OA**: **Age (years):** 70 ± 8.5, **mass (kg):** 69.5 ± 15.3, **height (m)**: 1.65 ± 0.1, **BMI (kg/m** ^**2**^ **):** 25.4 ± 4.9; **DASH**: 28.4 ± 15.9; **FIHOA:** 8.3 ± 5.6; **bilateral hand pain, *n* (%):** 13 ± 21.0; **number of painful joints, *n*:** 5.1 ± 3.9; **average hand pain, NRS (0–10):** 4.2 ± 2.2; **duration of pain, years:** 9.5 ± 9.1	**26 asymptomatic controls**: **Age (years):** 70 ± 11.5, **mass (kg):** 70.8 ± 15.2, **height (cm):** 1.66 ± 0.08, **BMI (kg/m** ^**2**^ **):** 25.4 ± 4.4	Grip and pinch isometric force steadiness tasks from a seated position using a digital hand and pinch meter dynamometer (Biometric Ltd). The target force was set at **50% MVC**.	**SD (%), CoV (%)**	↓ **Grip** force steadiness **(SD and CoV of force**; group main effects, *F*(1, 85) = 20.4; *p* < 0.0001 and *F*(1, 85) = 12.8; *p* < 0.01, respectively). ↓ **Pinch** force steadiness **(SD and CoV of force**; group main effects, *F*(1, 85) = 6.4; *p* < 0.05 and *F*(1, 85) = 6.4; *p* < 0.05, respectively).
Mista et al. (2018)	**19 chronic elbow pain patients,** 57% women, **age:** 41 ± 11 years, **weight:** 70.0 ± 16.4 kg, **height**: 166.3 ± 2.3 cm; **epicondyle PPT, N:** 15.8 ± 8.7, **tibialis anterior PPT, N:** 57.4 (20.2), **DASH (0–100):** 25.0 (15.6); **mean (SD) VAS scores (0–10) after isometric wrist extension**: **5% MVC:** 0.8 ± 0.2; **30% MVC:** 1.7 ± 0.4; **50% MVC:** 4.7 ± 0.4; **70% MVC:** 6.2 ± 0.4.	**21 asymptomatic controls,** 55% women, **age:** 37 ± 13 years, **weight:** 68.9 ± 12.5 kg, **height:** 161.5 ± 8.3; **epicondyle PPT, N:** 35.8 ± 11.5, **tibialis anterior PPT, N:** 71.5 ± 18.8; **DASH (0–100)**: 0.7 ± 2.4; **mean (SD) VAS scores (0–10) after isometric wrist extension**: **5% MVC:** 0.0 ± 0.2; **30% MVC:** 0.0 ± 0.4; **50% MVC:** 0.0 (0.4); **70% MVC:** 0.2 (0.4)	Isometric wrist extension using a six‐axis load cell transducer. Seated upright in a chair with their back resting against a backrest and the shoulder at 90°, participants performed two sets of isometric wrist extensions at **5%, 30%, 50% and 70% MVC**. Force was presented in real time on a computer screen.	**SD (N)**	No significant differences in force steadiness between patients and controls.
Miura & Sakuraba (2014)	**14 individuals with non‐specific low back pain**: **Age (year):** 21.1 ± 1.1, **height (cm):** 172.2 ± 5.5, **weight (kg):** 62.2 ± 4.4, **BMI:** 21:0 ± 1.9 **VAS scale pain intensity** >3/10.	**14 asymptomatic controls**: **Age (year):** 21.6 ± 2.3, **height (cm):** 173.8 ± 5.3, **weight (kg):** 65.5 ± 5.6, **BMI:** 21:7 ± 2.0	Isometric trunk extension using a force transducer (TU‐BR, TEAC). The target forces were performed in a random order with three trials at each of **2%, 5%, 10%, 15%, 20%, 30%, 50%, 70%, 80% and 90% of MVC** force. A visual display was positioned 50 cm away from the faces of the participants.	**SD (N)** **CoV (%)**	↓ Isometric trunk extension force steadiness in NSLBP (**CoV of force**; main effect of group, *F*(1, 26) = 4.698, *p* < 0.05. Specifically, CoV of the NSLBP group was higher than that of the controls at 30% and 50% MVC. No between‐ group differences for **force SD** *F*(1, 26) = 0.787, *p* = 0.383.
Muceli et al. (2011)	**19 people with chronic neck pain**: ***Exp 1 included nine women*** (age, mean ± SD: 40.4 ± 3.5 years; height, 171.1 ± 10.6 cm; body mass, 73.4 ± 10.6 kg); ***Exp 2 included 10 women*** (age, mean ± SD: 35.3 ± 7.5 years; height, 169.7 ± 7.4 cm; body mass, 72.2 ± 8.5 kg), different individuals for each experiment. ***Exp 1***: **onset (idiopathic trauma %trauma:** 33.3%; **length of history (years):** 13.6 (10.2); **NDI (0–50):** 14.8 ± 8.6; **average pain (VAS**: **0–10):** 4.4 ± 1.7; ***Exp 2***: **Onset (idiopathic trauma %trauma:** 80.0%; **length of history (years):** 7.6 ± 5.3; **NDI (0–50):** 24.8 ± 7.2; **average pain (VAS**: **0–10):** 6.0 ± 1.4; values are reported as **mean ± SD.**	**19 asymptomatic controls**: ***Exp 1 included nine healthy women*** (age, mean ± SD: 38.9 ± 10.5 years; height, 165.4 ± 8.2 cm; body mass, 63.6 ± 10.7 kg) ***Exp 2 included 10 healthy women*** (age, mean ± SD: 35.4 ± 8.9 years; height, 168.1 ± 5.1 cm; body mass, 66.5 ± 11.8 kg) as controls. Different individuals for each experiment. No history of pain.	Participants underwent isometric cervical flexion with their heads secured in a device that provided resistance through a force transducer. Seated with supported backs, knees and hips at 90 degrees, and torsos strapped to the seat, participants executed isometric neck contractions at either **15 N** (***Exp 1***) or **25% MVC** (***Exp 2***) for 10 seconds. The desired force output was shown on an oscilloscope placed 80 cm in front of them.	**CoV (%)**	In ***Exp 1***, ↓ cervical flexion force steadiness (**CoV**, main effect of group, *p* < 0.05). In ***Exp 2***, the CoV of force depended on the interaction between group and time (*p* < 0.05). People with chronic neck pain displayed ↓ force steadiness during submaximal sustained contractions compared to the control participants.
Overbeek et al. (2020)	**40 people with SAPS**: **Age, years (mean ± SD):** 50 ± 6.38, **female (*N*, %):** 23 (58%), **Right‐side dominance (*N*, %):** 35(88%), **dominant‐side measured/affected (*N*, %):** 25(63%), **duration of complaints (median, IQR):** 18 (12–29) **Clinical scores**: **VAS (0–100) for pain in rest:** 19 ± 20 **VAS (0–100) for pain during movement:** 39 ± 24 **Constant score:** 70 ± 13	**30 asymptomatic controls**: **Age, years (mean, SD):** 51 ± 5.71, **female (*N*, %):** 17 (57%), **right‐side dominance (*N*, %):** 25 (83%), **dominant‐side measured/affected (*N*, %):** 17(57%); **duration of complaints (median, IQR):** N/A; **VAS for pain in rest:** 2.1 ± 1.7; **VAS for pain during movement (0–100):** 2.0 ± 1.6 **Constant score:** 94 ± 4.1 Controls were recruited under a separate protocol.	Isometric shoulder abduction and adduction using a one‐dimensional force transducer at the wrist. Isometric shoulder ab‐ and adduction contractions from a standing position, facing a computer for force feedback, with the target arm in external rotation at the side attached to a one‐dimensional force transducer at the wrist. The target force was similar for both abduction and adduction and equal to **60% MVC** (defined as the lowest absolute value of the MVC in abduction or adduction).	**CoV (%), SD (N)** and **ApEn**	Better shoulder abduction force steadiness in people with SAPS **SD of force** (group difference: −0.006 N (CI: [−0.011 to −0.001], *p* = 0.013) and CV (group difference: *−*0.51 (CI: [−0.93 to −0.10], *p* = 0.016). No differences in magnitude of variability were observed during adduction. Patients with SAPS had lower ApEn‐values during the abduction task (−0.16, 95% CI: [−0.33 to −0.00], *p* = 0.048) and adduction task (−0.20, 95% CI: [−0.37 to −0.03], *p* = 0.024).
Testa et al. (2017)	**12 people with neck pain**: **Age (years):** 28.2 ± 6.2, **sex (% female):** 75, **weight:** 64.8 ± 7.7, **height:** 174 ± 11.6, **duration of pain (months):** 60.8 ± 63.7, **current pain intensity (NRS**: **0–10):** 5.1 ± 1.6, **NDI**: **(%):** 21.9 ± 7.5; ***SF‐36***: **physical:** 47.4 ± 5.5, **mental:** 47.8 ± 10.8; **TSK:** 30 ± 5 and **STAI:** 47 ± 6	**12 controls**: **Age (years):** 27.2 ± 6.5, **Sex (%female):** 75, **Weight:** 63.4 ± 11.9, **Height:** 169.3 ± 7.2	Submaximal isometric bilateral jaw clenching contractions using two force transducers. Participants were seated, hips and knees in 90° of flexion and their feet flat on the floor. Bilateral bite force was displayed in real time on the PC monitor. Contraction level: **10%, 30%, 50% and 70% MVC**. Contractions were performed both with and without visual feedback (total: 32 submaximal contractions).	**SD (%) and CoV (%)**	No differences in force steadiness between the two groups were observed for the **SD and CoV of force**.
Testa et al. (2015)	**10 people with neck pain**: **Age (years):** 28.9 ± 6.0, **sex (%female):** 70, **weight:** 64.8 ± 7.9, **height:** 175.2 ± 11.4, **duration of pain (months):** 67 ± 64.5, **current pain intensity (NRS**: **0–10):** 5.2 ± 1.5, **neck disability index (%):** 22.5 ± 7.1; ***SF‐36***: **physical:** 46.5 ± 5.3, **mental:** 47.6 ± 11.0; **TSK:** 29.2 ± 5.0; **STAI:** 48.7 ± 5.8	**10 controls**: **Age (years):** 27.2 ± 5.8, **sex (%female):** 70, **weight:** 63.9 ± 11.6, **height:** 170.0 ± 7.0	Submaximal isometric unilateral jaw clenching contractions. The experimental setup was same as in the study above (study 13). The experimental procedure then involved matching four force targets, representing **10%, 30%, 50% and 70% of MVC**. Participants performed these four contractions on both the right and left sides.	**SD (%)**	No differences in force steadiness between groups (*p* > 0.05).
Testa et al. (2018)	**12 individuals with TMD**: **Age (years):** 30.9 ± 7.4, **sex (%female):** 75, **weight:** 62.9 ± 10.5, **height**: 169.1 ± 8.7; **duration of pain (months):** 69.6 ± 37.6; **current pain intensity (NRS**: **0–10):** 1.6 ± 1.5; **side of the greatest pain (right, left, %):** 58.4, 41.6; **bilateral pain (%):** 75; **JPF (%):** 13.4 ± 5.4; **NDI (%):** 17.6 ± 7.0; **TSK:** 28.0 ± 530; **PCS:** 9.3 ± 11.1	**12 asymptomatic controls**: **Age (years):** 31.4 ± 7.9, **Sex (%female):** 75, **weight:** 66.0 ± 9.2, **height:** 171.9 ± 9.1	Submaximal isometric unilateral jaw clenching contractions. A flexible piezoresistive force transducer was used. For the patient group, the painful and non‐painful side were considered and in the case of bilateral symptoms, the side with the greatest pain intensity was considered the painful side (i.e. both sides were assessed). Same experimental set‐up as in the studies above (13 and 14). Participants performed contractions to match force targets representing **10%, 30%, 50% and 70% of MVC**. All participants performed the contractions with and without visual feedback (four contractions for feedback, no feedback conditions and both sides).	**SD (%)**	↓ Force steadiness was observed in people with myogenic TMD. The SD of force was dependent on the interaction between group and matched side (*χ* ^2^(1) = 4.21, *p* = 0.04). The multiple comparisons revealed that the SD of the most painful side of the patient group (SD = 4.89, 95% CI = 3.48–6.90) was significantly higher than the matched side (response ratio = 0.66, *t* ratio = −3.28, *p* < 0.01) and the unmatched side (response ratio = 1.34, *t* ratio = 2.69, *p* < 0.05) of the control group.
Wang et al. (2020)	**18 people with jaw pain**: **Age:** 32.78 ± 10.84, **gender:** 5 M, 13 F; **duration of pain (months):** 84.83 ± 71.94, *p* = 0.00; ***JFLS‐20***: **mastication (0–60):** 15.06 ± 8.23, *p* = 0.00; **jaw mobility (0–40):** 9.56 ± 5.90, *p* = 0.00; **emotional and verbal expression (0–80):** 6.44 ± 7.23, *p* = 0.00; ***GCPS***: **disability days (last 6 months):** 109.61 ± 66.64, *p* = 0.00; **characteristic pain intensity (0–100):** 44.63 ± 18.81, *p* = 0.00; **disability score (0–100):** 17.41 ± 19.35, *p* = 0.00; **OBC (0–100):** 27.56 ± 8.79, *p* = 0.00; **pain rating during the tasks (VAS**: **0–10) at low force:** 2.84 ± 2.17, **high force:** 4.47 ± 2.37. **Mean ± SD** values.	**16 asymptomatic controls**: **Age:** 31.31 (10.85), **gender:** 6 M, 10 F; **duration of pain (months):** 0.0 ± 0.0; ***JFLS‐20***: **mastication (0–60):** 0.25 ± 1.00; **jaw mobility (0–40):** 0.0 ± 0.0; **emotional and verbal expression (0–80):** 0.0 ± 0.0; ***GCPS***: **disability days (last 6 months):** 0.0 ± 0.0; **characteristic pain intensity (0–100):** 0.0 ± 0.0; **disability score (0–100):** 0.0 ± 0.0; **OBC (0–100):** 12.06 ± 9.69; **pain rating during the tasks (VAS**: **0–10) at low force:** 0.23 ± 0.32, **high force:** 0.57 ± 0.86. **Mean ± SD** values.	Submaximal isometric jaw clenching force steadiness tasks (custom‐built force transducer). Sitting position, with the bite plates held between the upper row and lower row of teeth in the mouth with the base of the device mounted on a table. Real‐time feedback on the force performance was provided. Two targets: a low‐force level (**2% MVC)** and moderate‐force level (**15% MVC**). Trials were split into four blocks of 24 trials. Each block consisted of 12 low‐force trials and 12 moderate‐force trials. Each participant therefore completed a total of 48 low‐force trials and 48 moderate‐force trials.	**SD (%), CoV (%)**	↓ Force steadiness in people with chronic jaw pain (**SD and CoV of force**; main effect of group: *p* = 0.03 for both).
Wang et al. (2018)	**17 people with jaw pain**: **Age:** 33.53 ± 12.65; **gender:** 5 M, 12 F; **duration of pain (years):** 9.56 ± 9.55, *p* < 0.05; ***JFLS‐20***: **mastication (0–10):** 2.26 ± 1.37, *p* < 0.05; **jaw mobility (0–10):** 2.34 ± 1.88, *p* < 0.05; **emotional and verbal expression (0–10):** 0.91 ± 1.65, *p* < 0.05; **global score (0–10):** 1.84 ± 1.48, *p* < 0.05; **OBC (0–100):** 31.94 ± 7.49, *p* < 0.05; ***GCPS***: **disability days (last 6 months):** 86.82 ± 53.09, *p* < 0.05; **characteristic pain intensity (0–100):** 42.84 ± 15.02, *p* < 0.05; **interference score (0–100):** 14.41 ± 17.02, *p* < 0.05; **low‐force level pre‐task pain intensity:**19.16 ± 15.8 and **high‐force level:** 17.77 ± 16.6 **(VAS**: **0–100)**. **Mean ± SD** values.	**19 asymptomatic controls**: **Age:** 29.16 ± 10.43, **gender**: 8 M, 11 F; **duration of pain (years):** 0.00 ± 0.00, ***JFLS‐20***: **mastication (0–10):** 0.05 ± 0.17; **jaw mobility (0–10):** 0.05 ± 0.23; **emotional and verbal expression (0–10):** 0.00 ± 0.00; **global score (0–10):** 0.04 ± 0.13; **OBC (0–100):** 15.18 ± 7.90; ***GCPS***: **disability days (last 6 months):** 0.05 ± 0.23; **characteristic pain intensity (0–100):** 1.40 ± 3.35; **interference score (0–100):** 0.00 ± 0.00; **pre‐task pain intensity at low**: 1.59 ± 3.1, **high**: 1.81 ± 3.0 **force level (VAS**: **0–100)**. **Mean ± SD** values.	Submaximal isometric jaw clenching force steadiness tasks. Supine position, with the bite plates held between the upper row and lower row of teeth in the mouth with the base of the force transducer device resting on the chest. A custom‐built LabVIEW software sampled the force signal and displayed it as a green bar on a magnetically shielded display, which was visible to the participant via a mirror, providing the participant with real‐time feedback on their force performance. Two runs of experimental trials, each consisting of five trials with alternating force production at low‐force level (**2% of MVC**) and high‐force level (**15% of MVC)**.	**SD (%), CoV (%)** and **ApEn**	↓ Force steadiness in the chronic jaw pain group (**SD and CoV of force**; main effect of group, *p* < 0.05). No differences in ApEn between the two groups (*p* > 0.05).

Abbreviations: ApEn, Approximate entropy; CI, confidence interval; CLBP, chronic low back pain; CLE, chronic lateral epicondylitis; CoV, coefficient of variation; DASH, disabilities of the arm, shoulder and hand; DYN, dynamic; F, females; FIHOA, functional index of hand osteoarthritis; GCPS, Graded Chronic Pain Scale; IAT, insertional Achilles tendinopathy; IQR, inter‐quartile range; ISO, isometric; JFLS‐20, Jaw Functional Limitation Scale; JPF, jaw pain and function; M, males; MVC, maximal voluntary contraction; N/A, not applicable; NDI, neck disability index; NRS, Numeric Rating Scale; NSLBP, non‐specific low back pain; OA, osteoarthritis; OBC, oral behavioural checklist; ODI, Oswestry Disability Index; PCS, Pain Catastrophizing Scale; PFP, patellofemoral pain syndrome; PPT, pressure pain threshold; RMS, root mean square; SAPS, sub‐acromial pain Syndrome; SD, standard deviation; SF‐36, 36‐Item Short‐Form Health Survey; SIS, shoulder impingement syndrome; STAI, State–Trait Anxiety Inventory; TF, target force; TMD, temporomandibular disorder; TSK, Tampa Scale of Kinesiophobia; VAS, Visual Analogue Scale.

**TABLE 2:** Characteristics of studies examining the effects of experimental pain on force steadiness.

Study	Population characteristics	Characteristics of pain	Force steadiness task and contraction intensity	Outcome measure	Results
Bandholm et al. (2008)	**Nine healthy individuals**: (age range: 22–37 years; mean age 27.7 years).	**Hypertonic saline (1 mL, 6%)** into the supraspinatus muscle. Shoulder pain **(VAS**: **0–10)** during **isometric** shoulder abduction contractions: **TF20:** 3.5 ± 1.5, **TF27.5:** 3.2 ± 1.6, **TF35:** 2.8 ± 1.4. Shoulder pain during **dynamic** shoulder abduction contractions: **TF20:** 2.9 ± 1.5, **TF27.5:** 3.1. ± 1.1, **TF35:** 3.8 ± 2.5.	Shoulder abduction contractions, seated position, pre‐, during‐ and post‐pain (after 15 min) on an isokinetic dynamometer (Kin‐Com KC 125 AP) and an oscilloscope 1 meter from the seat. Isometric (at 90°), concentric and eccentric. Dynamic contractions: 30–120° at 15°/s. Target forces: **20%, 27.5% and 35% MVC**. Two isometric and three dynamic contractions at each MVC level (isometric, 10 s hold).	**SD (%)** **CoV (%)**	↓ Isometric shoulder abduction force steadiness (**SD of force**; *F* = 6.5, *p* = 0.009). However, no changes in differences when measured as **CoV** (*F* = 2.9, *p* = 0.087). Experimental pain did not have any effect on shoulder abduction force steadiness during dynamic contractions (*p* > 0.05).
Del Santo et al. (2007)	**Eight asymptomatic volunteers** (5 M) were right‐handed participants (mean age 33.86 ± 11.51 years, range: 26–53).	**Ascorbic acid** in the ADM muscle belly and the BIC muscle (40 mg in 0.2 mL and 90 mg in 0.5 mL, respectively). Pain time duration of about 10 min. ADM/ average peak pain on the **VAS Scale (0–10) was** 6.25 ± 2.12 at about 1 min after injection. In BIC, chemically induced pain generated a cramp‐like, localized sensation at muscle belly. Nociceptive sensation lasted about 10 min and peaked at about 2 min after the end of injection (**average peak pain** was 5.75 ± 0.71; 0–10).	Fifth finger abduction (ADM) and elbow flexion (BIC) isometric contractions from a seated position. ADM's force was measured using a force transducer, with the fifth finger at 10° horizontal abduction. BIC measurement was done with the arm fixed, forearm supine and wrist neutral. Contractions: pre, during and post. Force level was set at **30% MVC** for both muscles (force transducer: FGP instrumentation, Les Clayes Sous Bois, France). Visual force feedback was provided on‐screen.	**CoV (%)**	↓ In force steadiness of the ADM and BIC muscles. Mean **CoV** of the ADM was significantly higher during pain (14.43% ± 0.30 SE) than in control (11.30% ± 0.20 SE) (*t* = 3.23, *p* = 0.008). Similarly, in the BIC, fluctuations of the force were higher during pain stimulation (**CoV** = 22.73% ± 0.52 SE) than in control (**CoV** = 19.56% ± 0.41 SE).
Farina et al. (2012)	**11 asymptomatic men** (age 25.1 ± 2.5 years)	**Hypertonic saline (0.5 mL, 5.8%)** into the right ADM muscle. The **NRS (0–10)** score for pain was 4.1 ± 1.8 at the beginning of the painful set of contractions and decreased, although not significantly, to 3.7 ± 2.2 by the end of the set of contractions.	Isometric fifth finger abduction: baseline, isotonic saline, hypertonic saline and post pain. Isometric contraction at **10% MVC** for 60 s. The target force output was displayed on a PC monitor located 1 m in front of the participant. Adjustable chair with the right arm extended in a force brace. The fifth finger was fixed in the isometric device for measurement of finger abduction forces.	**CoV (%)**	↓ Force steadiness after injection of hypertonic saline (**CoV of force**; *F* = 5.6, *p* < 0.01; SNK: *p* < 0.05).
Hirata et al. (2015)	**12 young asymptomatic volunteers** (7 M; age: 25 ± 4 years, height: 172 ± 11 cm; weight: 70 ± 13 kg)	**Hypertonic saline (1 mL, 5.8%)** injection into the right m. longissimus to induce low‐back pain. **Pain intensity (VAS**: **0–10):** 2.6 ± 0.4	Six series of submaximal isometric trunk extensions (before, during and after a painful or non‐painful injection). Each series comprised contractions at **5%, 10% and 20% of MVC** (each lasting 45 s). High‐sensitivity three‐dimensional force sensor (MC3A, AMTI, USA). Force feedback was displayed on a computer screen.	**SD (N)**	The variability of task‐related force was not altered by pain.
Martinez‐Valdes et al. (2021)	**15 asymptomatic volunteers** participated [age 26 (11) years, 9 M, 6 F].	**Hypertonic saline (0.5 mL, 5.8%)** into the TA muscle. Pain lasted for the full set of contractions, reaching a peak intensity (**NRS Scale**: **0–10)** of mean (SD) 6.3 (1.6) out of 10, 60 s after the hypertonic saline injection, with a range between 4.5 (2.2) and 3.3 (1.5) points after the first and last contraction respectively. Pain was felt at location of injection (TA muscle) and two participants also experienced referred pain to the lateral malleoli and dorsal region of the foot.	Participants were seated on a Biodex System 3 dynamometer and performed isometric ankle dorsiflexion contractions (90° ankle joint angle). They were asked to track sinusoidal torque trajectories at frequencies of **0.25** or **1 Hz** and amplitudes **of 5% or 10% MVC**. Under four conditions (baseline, isotonic, pain and post‐pain), they performed four sinusoidal contractions. Duration of each contraction: 40 s. Real‐time torque feedback was provided via a computer monitor.	**SD (%)** **CoV (%)**	↓ Torque variability during the pain condition at the fastest contraction rates when calculated both in terms of **SD** torque (frequency × condition interaction: *p* = 0.007, *η* ^2^ = 0.267) and **CoV** torque (Frequency × Condition interaction: *p* < 0.001, *η* ^2^ = 0.35).
Martinez‐Valdes et al. (2020)	**15 asymptomatic volunteers** participated in the experiments (age: mean (SD) 26 (3) years, 9 M, 6 F).	**Hypertonic saline (0.5 mL, 5.8%)** into TA muscle. The painful sensation lasted for the full set of contractions, reaching its peak: 6.3 (1.6) 1 min after the injection and ceasing completely within 500 s. Pain was consistently felt under the electrode grid by all participants (mainly under the 2nd, 3rd and 4th columns of the grid); however, three participants also experienced referred pain at the lateral malleoli. **NRS peak (0–10):** 6.34 ± 1.67 at 60 s.	Seated on the chair of a Biodex System 3, with the back flexed at 30°. The right leg (dominant side for all) was positioned over a support, the knee was flexed to 160° (with 180° representing full knee extension) and the foot was fixed to a footplate (90° ankle joint angle). Participants performed ramp‐hold contractions at **20%** or **70% MVC**. Two contractions were performed at each MVC level (four contractions in total, 10 s hold each). Real‐time torque feedback was provided via a computer monitor.	**SD (%)** **CoV (%)**	No differences in torque steadiness (average value across conditions: 2.2 (0.2)% at 20% MVC and 2.4 (0.2)% at 70% MVC, *p* = 0.76 and *p* = 0.20, respectively)
Mista et al. (2015)	**12 right‐handed asymptomatic individuals** (8 M, age 25 ± 5 years; height 171 ± 9 cm; weight 67 ± 10 kg, mean ± SD) participated in the study.	**Hypertonic saline (1 mL, 5.8%)** into the long head of the right BIC. **VAS (0–10) mean scores:** 3.5 ± 0.5 cm	Isometric elbow flexion contractions against a three‐dimensional force transducer. Contractions were performed using two types of visual feedback: (1) 1D displaying only the task‐related force component (i.e. elbow flexion force), and (2) 3D displaying the three force components. Contractions (20 s) using one‐dimensional and three‐dimensional feedback at **5%, 15% and 30% MVC** were performed pre‐, during and after experimental pain.	**Normalized SD, SampEn**	↓ In task‐related and tangential elbow flexion force variability during the one‐dimensional feedback (NK: *p* < 0.033). Post hoc analysis also revealed an increased force complexity during muscle pain and post‐pain compared with baseline (NK: *p* < 0.012).
Poortvliet et al. (2019)	**17 asymptomatic adults** (33 ± 6 years, 14 M)	**Hypertonic saline (0.25 mL, 5%)** into the infrapatellar fat pad of the test leg. Overall **pain intensity** reported during the contractions after hypertonic saline injection was as follows: 4.1 ± 1.3/10. (Majority with **NRS**: 0–10)	Isometric knee extension at **10% MVC**. Participants were supine, with the knee and hip flexed to 90°. A force gauge attached via an adjustable cable between the ankle and table was used.	**SD (N)**	↓ Isometric knee extension force steadiness Fluctuations of force around the target were as follows: no‐pain: 1.51 ± 0.49 and pain: 1.95 ± 0.66; *t‐*test: *p* < 0.001.
Rice et al. (2015)	**15 asymptomatic individuals** (mean age 28 years, range: 18–49 years; 8 F). One participant had an adverse effect. Data collected from 14.	**Hypertonic saline (1 mL, 5.8%)** into the infrapatellar fat pad. The average peak pain rating **(NRS**: **0–10)** following the injection was 5.5 ± 2.1 and lasted 18 ± 4 min after needle withdrawal.	Isometric knee extension, eccentric and concentric force steadiness contractions against a dynamometer (Biodex Medical Systems Inc, Shirley, New York, USA). For each contraction, the target was set at 1**0% MVC**. A monitor was used to show the actual knee extensor force to the participants.	**SD (N)**	↓ Knee force steadiness (**force SD**) during pain compared with baseline (*p* = 0.02) and with post‐pain (*p* < 0.001). The interaction effect between contraction and pain was not significant (*F*(4, 52) = 2.9; *p* = 0.06).
Salomoni et al. (2013)	**15 asymptomatic volunteers** (12 M, age, 27.1 ± 4.6 years, height, 175.1 ± 7.9 cm, weight, 71.9 ± 13.8 kg, mean ± SD)	**Hypertonic saline (1 mL, 5.8%)** into the medial part of the infrapatellar pad of the dominant leg. Peak in **VAS (0–10)** after 1.26 min, 4.52 ± 0.54 (SEM).	Isometric knee extension contractions (dominant leg) at **2.5%, 5%, 20%, 50% and 80% of MVC** force. A total of six series of contractions were performed (pre‐, during and post‐pain conditions). 3D forces were recorded using a high‐sensitivity six‐axis force sensor, yielding three‐force and three‐moment components (MC3A, AMTI, USA)	**CoV (%)**	↓ Knee force steadiness (**CoV)** of the task‐related force component compared with non‐painful assessments [RM‐anova: *F*(2, 8) > 10.8, *p* < 0.0003; NK: *p* < 0.0005].
Salomoni & Graven‐Nielsen (2012)	**15 asymptomatic individuals** (12 M, age 28.3 ± 6.5 years; height 175 ± 10 cm; weight 72.8 ± 12.7 kg; mean ± SD) with no known musculoskeletal disorder participated in this study	**Hypertonic saline (1 mL, 6%)** into one agonist muscle of each muscle group: TA for dorsiflexors, brachioradialis for elbow flexors, VM for knee extensors and GM for plantar flexors. **Peak pain (VAS**: **0–10, mean, SEM)**: **dorsiflexors (1.73 min):** 4.19 ± 0.61; **elbow flexors (1.16 min):** 4.49 ± 0.56; **knee extensors (1.22 min):** 4.16 ± 0.44; **plantar flexors (1.66 min):** 4.39 ± 0.5	Submaximal isometric force steadiness contractions of dorsiflexors, elbow flexors, knee extensors and plantar flexors at **2.5%, 20%, 50% and 70% of MVC** force. Six series of isometric contractions were performed before, immediately after the injection and after the cessation of any painful effects due to the injection. During all contractions, a computer screen provided a ramp‐and‐hold target force feedback corresponding to 2 s of ramp phase and 11 s of hold phase. A six‐axis force sensor was used to record 3D forces (MC3A 250, AMTI, USA)	**SD (N) CoV (%).**	No significant reductions in force steadiness (**SD of force**), except for a ↓ in knee extension force steadiness during the painful condition at 70% MVC (*F*(6, 78) = 2.4428, *p* = 0.0323; NK *p* = 0.0001). ↓ Force steadiness (**CoV of force**) (RM‐anova: *F*(2, 26)*–*28 > 4.82, *p* < 0.015; NK: *p* < 0.03) during pain for all muscle groups and, except for dorsiflexors, also higher than pre‐ and post‐injection conditions (NK: *p* < 0.03).
Smith et al. (2021)	**14 asymptomatic and recreationally active participants** (13 M, 1 F; mean ± SD: age: 25.3 ± 4.5 years; height: 1.78 ± 0.1 m; body mass: 73.9 ± 12.3 kg; physical activity: 5.6 ± 2.2 h/week) volunteered to participate in the present study.	**Hypertonic saline (1 mL, 5.8%)** into the middle third of the VL of the right leg. Pain intensity was measured with the **VAS Scale (0–10)**. **VAS mean:** 3.1 ± 1.0 **VAS peak:** 5.7 ± 1.7 **VAS onset:** 1.7 ± 1.3 **VAS time to peak, s:** 71 ± 24 **VAS duration, s:** 233 ± 60 **VAS area:** 759.8 ± 325.6	Participants engaged in isometric knee extension (right‐side) torque steadiness tasks using a Cybex HUMAC Norm isokinetic dynamometer at 15% and 20% MVC, with trials conducted both with and without force feedback. Target and actual torques were displayed on a computer. The study comprised two sessions: a control session with isotonic saline and an experimental session with hypertonic saline. In each session, participants completed six trials in a sequence of alternating feedback conditions: feedback, no feedback, feedback, no feedback, feedback and no feedback.	**ApEn SampEn**	The presence of pain did not influence torque complexity, measured as ApEn or SampEn (*p* > 0.05).
Yavuz et al. (2015)	**9 of 11 asymptomatic individuals** (9 M; age: 25.1 (2.5) years) for the ADM muscle and **7 of 12 asymptomatic individuals** (7 M; age 24.2 (2.1) years) for the TA muscle. Two participants were excluded because of the limited number of motor units identified.	**Hypertonic saline (0.5 mL, 5.8%)** into the ADM and TA muscles. **ADM (pain**: **NRS**: **0–10):** 4.4 (2.1) **TA (pain**: **NRS**: **0–10):** 3.5 (1.2)	In **Experiment 1**, participants were seated with the arm and first four digits secured, and the fifth digit attached to a load cell to measure isometric abduction force. They performed a 60‐s isometric abduction of the fifth digit at 10% MVC under three conditions: baseline, isotonic saline and hypertonic saline. In **Experiment 2**, participants, with their foot in an isometric force brace, executed a 4‐min isometric dorsiflexion at 25% MVC. After a 20‐min rest, one leg was treated with hypertonic saline (to induce discomfort) and the other with isotonic saline. Following the infusion, the isometric dorsiflexion was repeated, with visual force feedback provided via an oscilloscope in both experiments.	**CoV (%)**	↓ Force steadiness after the injection of hypertonic saline for both muscles (ADM and TA) when measured as SD of force (*p* < 0.01 for both) and CoV of force (*p* < 0.05 for both).

Abbreviations: 1D, one dimensional; 3D, three dimensional; ADM, Abductor digiti minimi; BIC, biceps brachii; CoV, coefficient of variation; F, females; GM, gastrocnemius medialis; M, males; MVC, maximal voluntary contraction; NRS, Numeric Rating Scale; RM‐ANOVA, repeated measures analysis of variance; SampEn, sample entropy; SD, standard deviation; SE, standard error; TA, tibialis anterior; TF, target force; VAS, Visual Analogue Scale; VM, vastus medialis.

#### 3.2.1 Clinical pain studies

Nineteen studies investigated the impact of clinical pain on force steadiness. These involved a total of 756 participants, of whom 402 were experiencing musculoskeletal pain and the rest were asymptomatic controls. Specifically, from these 19 studies, 5 assessed force steadiness at the jaw (*n* = 69; chronic neck pain, CNP; chronic jaw pain; and temporomandibular disorders, TMDs) (Testa et al., 2015; Testa et al., 2017; Testa et al., 2018; Wang et al., 2018; Wang et al., 2020), 3 at the shoulder (*n* = 76; subacromial impingement syndrome (Bandholm et al., 2006; Camargo et al., 2009; Overbeek et al., 2020) and another 3 for the lower back (*n* = 49, chronic low back pain; CLBP) (Arvanitidis et al., 2022; Arvanitidis et al., 2024; Miura & Sakuraba, 2014). Additionally, two studies assessed force steadiness at the neck (*n* = 28; CNP) (Falla et al., 2010; Muceli et al., 2011), another two at the knee (*n* = 50, osteoarthritis) (Ferreira et al., 2021; Hortobágyi et al., 2004) and another two at the hand or wrist (*n* = 81; osteoarthritis) (Magni et al., 2021; Mista et al., 2018). Finally, one study focused on the ankle (*n* = 34; insertional Achilles tendinopathy) (Crowley et al., 2022) and another one on the elbow (*n* = 15; chronic lateral epicondylitis). The average age of participants ranged from 21 to 70 years, while the pain intensity scores reported by the patients ranged from 0.8 to 7.1 out of 10 (Numeric Rating Scale, NRS; or Visual Analogue Scale; VAS).

Thirteen of these studies reported a significant decline in force steadiness in the presence of pain (Arvanitidis et al., 2022; Arvanitidis et al., 2024; Bandholm et al., 2006; Chen et al., 2023; Falla et al., 2010; Ferreira et al., 2021; Hortobágyi et al., 2004; Magni et al., 2021; Miura & Sakuraba, 2014; Muceli et al., 2011; Testa et al., 2018; Wang et al., 2018; Wang et al., 2020). However, five studies observed no significant differences between those with and without pain (Camargo et al., 2009; Crowley et al., 2022; Mista et al., 2018; Testa et al., 2015; Testa et al., 2017), and one study (Overbeek et al., 2020) observed greater force steadiness in those with shoulder pain during shoulder abduction contractions, whereas no significant difference was observed during shoulder adduction. Notably, two of the five studies (Testa et al., 2015; Testa et al., 2017) which found no significant differences specifically investigated jaw clenching steadiness in individuals with neck pain; however, when the same research group specifically assessed jaw clenching force steadiness in a cohort of individuals with TMDs, that is, where the pain was task specific, a significant difference was observed.

#### 3.2.2 Experimental pain studies

Thirteen studies involving a total of 174 participants were evaluated to investigate the effect of experimental pain on force steadiness. For 12 studies, hypertonic saline was the experimental pain model used, and one further study (Del Santo et al., 2007) used ascorbic acid. Four studies assessed the effect of experimental pain on knee force steadiness (*n* = 61) (Poortvliet et al., 2019; Rice et al., 2015; Salomoni et al., 2013; Smith et al., 2021) and two on the ankle (*n* = 30) (Martinez‐Valdes et al., 2021, 2020). One study assessed the low back (*n* = 12) (Hirata et al., 2015), one the shoulder (*n* = 9) (Bandholm et al., 2008), one the elbow (n = 12) (Mista et al., 2015) and one the fifth finger (*n* = 11) (Farina et al., 2012). Additionally, three of these studies investigated the effect of experimentally induced pain on force steadiness in various regions. One study evaluated this at the elbow and fifth finger (*n* = 8) (Del Santo et al., 2007), another one at the ankle, elbow and knee (*n* = 15) (Salomoni & Graven‐Nielsen, 2012) and a third one at the fifth finger and ankle (*n* = 16) (Yavuz et al., 2015). Most of these studies (10 of the 13) (Bandholm et al., 2008; Del Santo et al., 2007; Farina et al., 2012; Martinez‐Valdes et al., 2021; Mista et al., 2015; Poortvliet et al., 2019; Rice et al., 2015; Salomoni et al., 2013; Salomoni & Graven‐Nielsen, 2012; Yavuz et al., 2015) observed reductions in force steadiness when experimental pain was induced. In contrast, three studies found no differences in force steadiness (Hirata et al., 2015; Martinez‐Valdes et al., 2020) or torque complexity (Smith et al., 2021) between conditions with and without pain for the same individuals. However, the SD of torque data provided by Smith et al. (2021), which was included in the meta‐analysis, revealed that force steadiness is reduced in the presence of experimental pain when quantified with this variable. The average age of participants ranged from 22 to 53 years, while the peak pain intensity scores reported by the patients during the task ranged from 2.6 to 6.3 out of 10 (NRS or VAS scales).

### 3.3 Risk of bias

Comprehensive summaries of the risk of bias scores are presented in Tables 3 and 4 respectively. In the assessment of risk of bias across the 19 clinical pain studies, the scores varied from 4 to 9 out of a possible 9. The quality of studies was rated as ‘*poor’* for 10, *‘good’* for 8 and *‘fair’* for 1 (Table 3). The most common reasons for *‘poor’* ratings were from potential biases introduced by not matching participants on age and/or gender as part of the study methodology, and not reporting full participant recruitment information. The assessment was also performed across 13 experimental pain studies with the scores ranging between 5 and 9 out of 9. The quality was rated as *‘good’* in eight of the studies, *‘fair’* in three and *‘poor’* in two (Table 4).

**TABLE 3:** Risk of bias of clinical pain studies.

Study	Selection	Comparability	Exposure	Total	Quality
Arvanitidis et al. (2022)	★★★		★★★	6	Poor
Arvanitidis et al. (2024)	★★★		★★★	6	Poor
Bandholm et al. (2006)	★	★★	★★★	6	Poor
Camargo et al. (2009)	★★	★★	★★★	7	Fair
Chen et al. (2023)	★		★★★	4	Poor
Crowley et al. (2022)	★★★	★★	★★★	8	Good
Falla et al. (2010)	★		★★★	4	Poor
Ferreira et al. (2021)	★★★★		★★★	7	Poor
Hortobágyi et al. (2004)	★★★★	★★	★★	8	Good
Magni et al. (2021)	★★★	★★	★★★	8	Good
Mista et al. (2018)		★★	★★★	5	Poor
Miura & Sakuraba (2014)	★		★★★	4	Poor
Muceli et al. (2011)	★★★★		★★★	7	Poor
Overbeek et al. (2020)	★★★★	★★	★★	8	Good
Testa et al. (2017)	★★★★	★★	★★★	9	Good
Testa et al. (2015)	★★★★	★★	★★★	9	Good
Testa et al. (2018)	★★★	★★	★★★	8	Good
Wang et al. (2020)	★★★★	★	★★★	8	Good
Wang et al. (2018)	★★		★★★	5	Poor

**TABLE 4:** Risk of bias of studies of experimental pain studies.

Study	Selection	Comparability	Exposure	Total	Quality
Bandholm et al. (2008)	★★	★	★★★	6	Fair
Del Santo et al. (2007)	★★		★★★	5	Poor
Farina et al. (2012)	★	★	★★★	5	Poor
Hirata et al. (2015)	★★	★★	★★★	7	Fair
Martinez‐Valdes et al. (2021)	★★★	★★	★★★	8	Good
Martinez‐Valdes et al. (2020)	★★★	★★	★★★	8	Good
Mista et al. (2015)	★★★	★	★★★	7	Good
Poortvliet et al. (2019)	★★★	★	★★★	7	Good
Rice et al. (2015)	★★	★	★★★	6	Fair
Salomoni et al. (2013)	★★★	★★	★★★	8	Good
Salomoni & Graven‐Nielsen (2012)	★★★	★★	★★★	8	Good
Smith et al. (2021)	★★★	★★	★★★	8	Good
Yavuz et al. (2015)	★★★	★★	★★★	8	Good

### 3.4 Results of syntheses and certainty of evidence

Meta‐analyses for both clinical and experimental pain studies were conducted using the CoV and SD of force measures. However, since some studies utilized either the CoV or the SD measure, but not both, it was not feasible to incorporate all studies into both meta‐analyses. As a result, some studies could be included in either the CoV‐based meta‐analysis or the SD‐based meta‐analysis, but not both.

#### 3.4.1 Force steadiness in the presence of clinical pain

**FIGURE 2:**
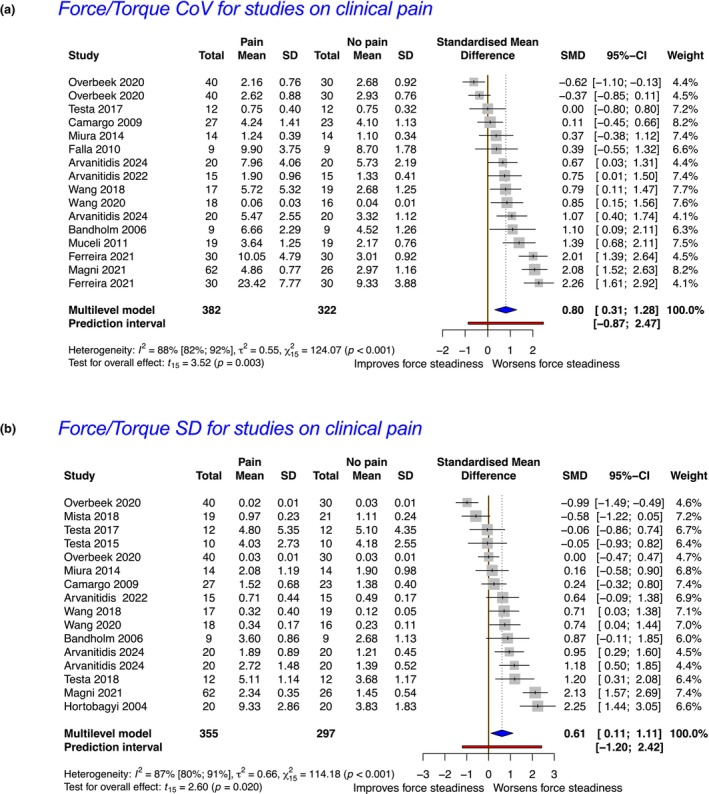
Meta‐analyses on the effect of clinical pain on force steadiness during different submaximal voluntary contractions. The mean ± SD of each outcome measure and sample size for each group are reported, alongside the standardized mean difference and 95% confidence interval (95% CI). The analysis is divided into two segments, (a) focuses on the coefficient of variation (CoV) of force/torque, while (b) is based on the SD of force/torque. All heterogeneity measures and overall effect size tests are reported below each meta‐analysis. The prediction interval is also depicted as a red line within the graph. The forest plots are organized in ascending order of their effect sizes.

The results of the meta‐analysis that included 13 of the total 19 studies with clinical pain, involving 382 participants with pain and 322 without, demonstrated that there was a significant overall effect of musculoskeletal pain on the CoV of force, indicating that force steadiness is impaired in the presence of pain (SMD = 0.80 [95% CI: 0.31–1.28], *I* ^2^ = 88% [95% CI: 82%–92%], *τ* ^2^ = 0.55, *Q* = 124.07, *t*_15_ = 3.52, *p* = 0.003). Similarly, the results of the meta‐analysis that included 14 of the total 19 studies with clinical pain, involving 355 participants with pain and 297 without, demonstrated that there was a significant overall effect of musculoskeletal pain on the SD of force, indicating impaired force steadiness in the presence of pain (SMD = 0.61 [95% CI: 0.11–1.11], *I* ^2^ = 87% [95% CI, 80%–91%], *τ* ^2^ = 0.66, *Q* = 114.18, *t*_15_ = 2.60, *p* = 0.020). The results of the forest plots with meta‐analyses are presented in Figure 2.

One study was excluded from both meta‐analyses because related data could not be extracted (Crowley et al., 2022). This study did not observe any differences in plantar flexor steadiness (measured as CoV of force) in people with insertional Achilles tendinopathy compared to controls. In the CoV‐based meta‐analysis, four studies were not included because they quantified force steadiness using the SD of force. Among these, two observed reductions in force steadiness in people with TMD (Testa et al., 2018) and knee osteoarthritis (Hortobágyi et al., 2004) compared to controls, while the other two found no significant differences in individuals with chronic elbow pain (Mista et al., 2018) or people with CNP (Testa et al., 2015). Conversely, in the SD‐based meta‐analysis, three studies were excluded because they only used CoV of force as the outcome measure with all observing significant reductions in force steadiness in people with CNP (Falla et al., 2010; Muceli et al., 2011) and PFP (Ferreira et al., 2021), which is in line with the findings of the meta‐analysis.

#### 3.4.2 Force steadiness in the presence of experimental pain

**FIGURE 3:**
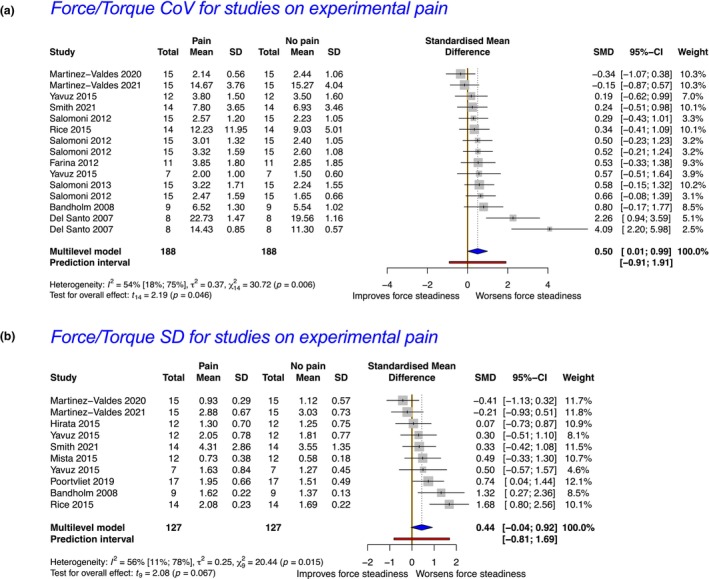
Meta‐analyses on the effect of experimental pain on force steadiness during different submaximal voluntary contractions. The mean ± SD of each outcome measure and sample size for each group are reported, alongside the standardized mean difference and 95% confidence interval (95% CI). The analysis is divided into two segments, (a) focuses on the coefficient of variation (CoV) of force/torque, while (b) is based on the SD of force/torque. All heterogeneity measures and overall effect size tests are reported below each meta‐analysis. The prediction interval is also depicted as a red line within the graph. The forest plots are organized in ascending order of their effect sizes.

The results of the meta‐analysis that included 10 out of the total 13 studies on experimental pain, involving 188 participants measured under both pain and no pain conditions, demonstrated that there was a significant overall effect of experimental musculoskeletal pain on the CoV of force indicating worse force steadiness in the presence of pain (SMD = 0.50 [95% CI: 0.01–0.99], *I* ^2^ = 54% [95% CI: 18%–75%], *τ* ^2^ = 0.37, *Q* = 30.72, *t*_14_ = 2.19, *p* = 0.046). However, the results of the meta‐analysis that included 9 out of the total 13 studies with experimental pain, involving 127 participants measured under both pain and no pain conditions, demonstrated that there was not a significant overall effect of experimental musculoskeletal pain on the SD of force (SMD = 0.44 [95% CI: −0.04 to 0.92], *I* ^2^ = 56% [95% CI: 11%–78%], *τ* ^2^ = 0.25, *Q* = 20.44, *t*_9_ = 2.08, *p* = 0.067). The results of the forest plots with meta‐analyses are presented in Figure 3.

Three studies were excluded from the CoV‐based meta‐analysis. Among these, two did not observe any differences in elbow flexion (Mista et al., 2015) and trunk extension (Hirata et al., 2015) force steadiness between the experimental pain and baseline conditions, while the other one showed that knee extension force fluctuations were higher in the presence of experimentally induced knee pain (Poortvliet et al., 2019). Four studies were excluded from the SD‐based meta‐analysis. Among these studies, three showed that force steadiness is reduced in the presence of experimentally induced knee (Salomoni et al., 2013), fifth finger (Farina et al., 2012) and fifth finger and elbow pain (Del Santo et al., 2007). The last one investigated the effect of experimentally induced pain on the force steadiness of multiple muscles and observed reductions in force steadiness only for the knee at higher forces (Salomoni & Graven‐Nielsen, 2012).

#### 3.4.3 Alternative measures of force control

There was inadequate data to conduct meta‐analyses on studies involving other measures of force control. Specifically, in the studies with clinical pain, only two used ApEn as a measure of force complexity with one of them showing that people with sub‐acromial pain syndrome had lower ApEn values during shoulder abduction and adduction tasks (i.e. a smoother force pattern with less variability) compared to the controls (Overbeek et al., 2020), while the other did not observe any differences for ApEn values during a jaw clenching task in people with and without jaw pain (Wang et al., 2018). These studies were included in our meta‐analysis as they also assessed steadiness using the CoV or SD of torque. However, another study with clinical pain was not included in any meta‐analysis because it only used RMS and SampEn to quantify changes in force control between the two groups. This study showed that RMS was larger in people with CLE, while no differences were observed in terms of the complexity of the force signal (measured using SampEn) (Chen et al., 2023).

From the experimental pain studies, only two used alternative measures of force control to assess the effect of experimental pain on force control. One used both ApEn and SampEn to assess changes in isometric knee extension force control (Smith et al., 2021), while the other only the latter during elbow flexion contractions (Mista et al., 2015), with both studies not observing significant differences in these variables during the painful and non‐painful states.

#### 3.4.4 Certainty of evidence – Sensitivity analyses and GRADE

Influence analyses on the main meta‐analyses for both clinical and experimental studies, focusing on force/torque CoV and SD, did not identify any potential outliers, except for the SD‐based meta‐analysis in experimental pain studies, where one study (Rice et al., 2015) was detected as a potentially influential (Data S4). This study was removed as part of the sensitivity analysis to explore its influence on the overall result as described below. Additionally, the sensitivity analyses included: (i) removal of one study at a time, (ii) removal of studies that did not provide visual force feedback and (iii) removal of studies classified as having poor quality.

##### Clinical pain studies

Sensitivity analyses indicated that the overall results of the meta‐analyses for both force/torque CoV and SD were not influenced by the removal of any single study or by excluding data from studies that did not provide force visual feedback (Data S4). However, removing studies of poor quality altered the overall result, showing no differences in force steadiness in the presence of clinical pain. This change is likely due to the removal of the majority of studies from the meta‐analysis (eight and six studies, respectively). This likely does not provide an accurate representation, as many studies were rated poor, primarily due to a lack of information on the comparability of groups within the methodology, which was only reported in the results section.

##### Experimental pain studies

Removing the study (Rice et al., 2015) that could potentially influence the SD‐based meta‐analysis resulted in a similar outcome to the meta‐analysis that included all studies. Removing each study one at a time had a significant impact on the CoV‐based meta‐analysis, usually showing no changes in force steadiness in the presence of experimentally induced pain. For the SD‐based meta‐analysis, the result of no differences was maintained most of the time, but the overall effect became significant when two studies were removed (Martinez‐Valdes et al., 2021, 2020). Excluding force/torque SD data from studies that did not provide visual feedback did not alter the overall result of the main meta‐analysis. However, the CoV‐based meta‐analysis result changed when these data were removed, suggesting that experimentally induced pain does not influence force steadiness. Further exploration identified one study as potentially influential in the model; when this study was removed, the same result as the main meta‐analysis was observed. Lastly, removing two studies of poor quality (Del Santo et al., 2007; Farina et al., 2012) did not alter the overall result for the CoV‐based meta‐analysis, while no studies could be removed from the SD‐based meta‐analysis since none were classified as having poor quality.

**FIGURE 4:**
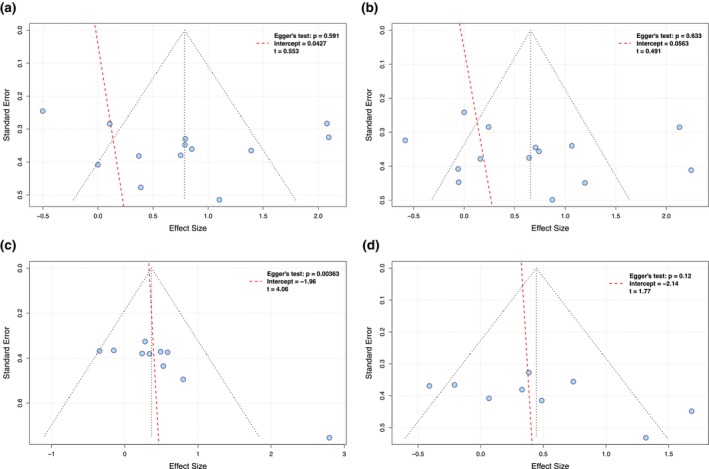
Funnel plots of the four meta‐analyses performed alongside the results of the Egger regression test. Funnel plots are presented for (a) torque CoV for clinical pain studies, (b) torque SD for clinical pain studies, (c) torque CoV for experimental pain studies and (d) TORQUE SD for experimental pain studies. The blue dots in each scatterplot represent the values from individual studies, with effect size on the *x*‐axis and standard error on the *y*‐axis. The middle dotted line represents the overall effect size, and the side dotted lines form a triangle indicating the 95% confidence interval for the expected distribution of studies in the absence of publication bias. The dotted red line represents Egger's regression line, used to test for potential sources of publication bias. Egger's test values of *p* < 0.05 indicate potential publication bias.

The evaluation of the certainty of evidence with GRADE was conducted independently for studies with clinical and experimental pain, based on the outcome used. This assessment was performed on studies using the CoV and SD of force as measures of force steadiness, due to the limited number of studies using alternative metrics. Any sources of publication bias were assessed by visually inspecting funnel plots and using Egger's regression tests, as shown in Figure 4.

The results showed that for the clinical pain studies, funnel plots were quite symmetrical, and Egger's regression test did not reveal any potential sources of publication bias (torque CoV: *p* = 0.591, intercept = 0.0427, *t* = 0.553; torque SD: *p* = 0.633, intercept = 0.0563, *t* = 0.491; Figure 4a, b, respectively). In contrast, for the experimental pain studies, the funnel plot for Torque CoV (Figure 4c) suggested potential sources of publication bias, as indicated by the clustering of dots around the 0.4 level of standard error and confirmed by Egger's regression test (*p* < 0.01, Intercept = −1.96, *t* = 4.06). However, the funnel plot for torque SD (Figure 4d) did not reveal any significant publication bias (*p* = 0.12, intercept = −2.14, *t* = 1.77).

Considering the above and the other domains assessed with GRADE, the findings from the meta‐analysis indicate, with moderate and low levels of evidence strength, that force steadiness is impaired in the presence of clinical pain when measured using force CoV and SD respectively. Additionally, it indicates with very low strength of evidence that force steadiness is impaired in the presence of experimental pain when quantified as the CoV of force, but not when quantified using the SD of force. The summary of findings is presented in Table 5.

**TABLE 5:** Summary of the overall certainty of evidence for each outcome, differentiated between clinical and experimental pain studies assessing the influence of pain on force/torque steadiness using the GRADE approach.

Studies included in each GRADE assessment	Outcome/finding	Confidence	Reason for downgrade or upgrade
Studies with clinical musculoskeletal pain			
Arvanitidis et al. (2022), Arvanitidis et al. (2024), Bandholm et al. (2006), Camargo et al. (2009), Falla et al. (2010), Ferreira et al. (2021), Magni et al. (2021), Miura & Sakuraba (2014), Muceli et al. (2011), Overbeek et al. (2020), Testa et al. (2017), Wang et al. (2018), Wang et al. (2020), **Crowley et al.** (2022)	Reduced force steadiness (force CoV)	Moderate	↓ Study limitations (mainly due to group comparability) ↑↑ Large magnitude of effect and derived from direct evidence
Arvanitidis et al. (2022), Arvanitidis et al. (2024), Bandholm et al. (2006), Camargo et al. (2009), Hortobágyi et al. (2004), Magni et al. (2021), Mista et al. (2018), Miura & Sakuraba (2014), Overbeek et al. (2020), Testa et al. (2017, 2015, 2018), Wang et al. (2018), Wang et al. (2020)	Reduced force steadiness (force SD)	Low	↓ Inconsistency (sensitivity analysis had minimal impact on heterogeneity, with some p‐values approaching statistical insignificance) ↑ Moderate magnitude of effect and derived from direct evidence
Studies with experimental musculoskeletal pain			
Bandholm et al. (2008), Del Santo et al. (2007), Farina et al. (2012), Martinez‐Valdes et al. (2021, 2020), Rice et al. (2015), Salomoni & Graven‐Nielsen (2012), Salomoni et al. (2013), Smith et al. (2021), Yavuz et al. (2015)	Reduced force steadiness (force CoV)	Very low	↓ Inconsistency, ↓ Imprecision, ↓ Publication bias Significant change in results when sensitivity analysis is performed; wide confidence intervals (CIs) and small sample size studies; asymmetry of funnel plots and Egger's test.
Bandholm et al. (2008), Hirata et al. (2015), Martinez‐Valdes et al. (2021, 2020), Mista et al. (2015), Poortvliet et al. (2019), Rice et al. (2015), Smith et al. (2021), Yavuz et al. (2015), **Salomoni & Graven‐Nielsen (** **2012** **)**	No changes in force steadiness (force SD)	Very low	↓ Imprecision (wide CIs, a few studies of small sample size)

*Note*: Studies highlighted in bold were excluded from the meta‐analysis for the specified outcome.

## 4 DISCUSSION

We investigated the influence of clinical and experimentally induced musculoskeletal pain on force steadiness. Integrating data from 32 studies, our analysis indicated that clinical musculoskeletal pain is associated with a decrease in force steadiness, as indicated by large and moderate effects on the CoV and SD of force. Experimental pain was associated with a reduction in force steadiness only as measured by the CoV of force (moderate effect), and not in the SD of force.

### 4.1 Does clinical pain influence force steadiness?

Our findings confirm that clinical musculoskeletal pain is associated with reduced force steadiness and align with a previous systematic review's findings indicating that peripheral musculoskeletal conditions are also associated with increased force CoV (Pethick et al., 2022). The high heterogeneity observed is likely attributed to differences in participant characteristics, the diverse nature, severity and duration of pain, methodological differences and the spectrum of musculoskeletal conditions studied. However, sensitivity analysis confirmed our findings' robustness, showing that no single study's exclusion changed the overall result (Data S4).

While most studies support that clinical pain is associated with reduced force steadiness, some exceptions were observed. Two studies (Testa et al., 2015, 2017) on jaw clenching force steadiness in people with CNP found no significant between‐group differences. However, assessments of people with TMD by the same authors showed reduced force steadiness in the patient group (Testa et al., 2018). This indicates that experiencing pain at the assessed location is crucial for such observations. Additionally, variations in force steadiness could be attributed to the assessed force level. Some studies examined a broad range of low‐to‐high submaximal forces, whereas some examined a single submaximal level. Deficits reported at specific force levels by certain studies (Bandholm et al., 2006; Miura & Sakuraba, 2014) imply that force steadiness deficits could be force‐level specific.

### 4.2 Does experimental pain influence force steadiness?

This review also revealed that experimental musculoskeletal pain is associated with reduced force steadiness when measured as the CoV of force. The meta‐analyses showed lower heterogeneity and smaller, more consistent effect sizes. This consistency is likely due to the use of similar pain models and the assessment of comparable anatomical regions in many studies. One study had a notably larger effect size, likely due to its unique use of ascorbic acid to induce pain (Figure 3a). However, the p‐values from both the CoV‐ and SD‐based meta‐analyses, along with insights from the sensitivity analyses in Data S4, suggest that the removal of some of the individual studies significantly influenced the overall effect. Therefore, these findings should be interpreted with caution.

The absence of significant differences in force SD may be due to the characteristics of the force steadiness outcome measures. The CoV quantifies variability relative to the mean force output, reflecting proportional changes, while SD, as an absolute measure, is inherently sensitive to individual strength differences. This distinction is more apparent in data synthesis across multiple studies, where variability in baseline participant strength can substantially influence SD outcomes. Moreover, the measurement unit heterogeneity (e.g. Newtons vs. percentages) across studies might confound comparisons, although this issue was mitigated in our analysis by using SMD for meta‐analytic calculations. These factors, coupled with the sample sizes and the number of studies included, could explain the observed discrepancies between CoV and SD findings. Interestingly, although in experimental pain the effect size for torque SD was not significant, the effect sizes for both torque SD and CoV were very similar. This similarity suggests that the overall trend towards impaired force steadiness in the presence of experimental pain is consistent across these measures. However, interpreting CoV results necessitates caution; sensitivity analysis indicated that excluding certain studies substantially alters outcomes, underscoring careful result interpretation and advocating for using both CoV and SD to assess force steadiness in people with pain (Data S4).

### 4.3 Differences between clinical and experimental pain

The findings suggest that the force steadiness deficits are less marked during experimental pain than in clinical pain conditions, a disparity likely stemming from the fundamental differences between these two pain types. Clinical pain, characterized by its multidimensional and persistent nature, encompasses physical, psychological and emotional elements, along with extensive neurophysiological changes. As clinical pain becomes chronic, significant modifications occur in the nervous system, including alterations in brain structure and function, particularly in areas involved in emotional and sensory processing (Sibille et al., 2016). These alterations include reductions in grey matter volume and white matter integrity, changes in neurotransmitter activity and reduced descending inhibition (Sibille et al., 2016). Such comprehensive changes across multiple levels of the nervous system, coupled with alterations in muscle structure, including atrophy, fatty tissue infiltration and fibre type changes, documented in people with chronic pain, are more likely to alter neuromuscular control than experimental pain (Matheve et al., 2023).

Experimentally induced pain, associated with transient sensitization of nociceptive pathways, differs from clinical pain due to its temporary nature and individuals' knowledge that the discomfort will cease. This understanding may affect their pain perception and response, contrasting with the unpredictability of clinical pain. Previous work further supports this, indicating that experimental pain does not replicate the changes in motor neuron excitability observed in clinical pain conditions across the neuromuscular pathway (Sanderson et al., 2021). In acute experimental pain, individuals might employ more strategies, such as changing movement and muscle patterns, to compensate for pain, which could explain why force deterioration appears less pronounced in experimental studies (Devecchi et al., 2023).

### 4.4 Physiological explanation of the observed findings

Pain likely impairs muscle force control through altered sensory input from the affected area and/or central changes (Ager et al., 2020). Structural alterations in brain areas essential for proprioceptive input processing or muscle composition changes may also contribute to impaired muscle force control (Pijnenburg et al., 2014; Sterling et al., 2001). These sensory integration changes can alter the efferent response, thereby influencing muscle recruitment strategies employed by individuals to perform muscle contractions.

Alterations in force steadiness may be linked to changes in motor unit behaviour. Reduced modulation in the discharge rates of motor units in the sternocleidomastoid muscle in women with CNP has been observed, indicating a change in neural drive to muscles in the presence of pain (Falla et al., 2010). Additionally, increased motor unit recruitment and firing rates have been observed in early knee osteoarthritis (Ling et al., 2007) and in women with PFP (Gallina et al., 2018) respectively. Experimentally induced knee pain influences force steadiness in healthy individuals (Rice et al., 2015), and notably, changes in motor unit discharge patterns have also been reported in the presence of experimental pain within the same region (Poortvliet et al., 2019).

Recent studies have indicated that the effective neural drive predominantly operates in the low‐frequency band (<10 Hz), reflecting the common synaptic input to the motor unit pool, essential for force generation (Farina et al., 2014; Negro et al., 2009). Since muscle force control relies on the amplitude of these oscillations, increased amplitude fluctuations observed in pain conditions may indicate greater variability in synaptic input to α motor neurons. Given the α motor neuron's role in integrating complex inputs and the interplay between proprioceptive and nociceptive signals, the physiological mechanism by which musculoskeletal pain influences force control may vary among different pain types and individuals (Hug et al., 2023).

### 4.5 Clinical relevance

Impaired force steadiness may lead to suboptimal tissue loading, where a disrupted sense of body position may result in the application of excessive forces in unfavourable positions during movement. This abnormal mechanical stress on tissues can activate nociceptors and cause tissue strain, potentially contributing to the development of additional pain or injuries and the perpetuation of existing symptoms. These impairments likely have clinical relevance and could become targets for treatment for people experiencing musculoskeletal pain.

### 4.6 Limitations

This review's limitations include the predominance of observational studies in the meta‐analyses, which hinders causal inference between musculoskeletal pain and decreased force steadiness. Factors such as reduced activity, medication, sleep and psychological conditions may also influence force steadiness. The relatively small sample sizes of the included studies, particularly in experimental pain studies, also pose a challenge to the robustness of the evidence. Nonetheless, within‐participant comparisons mitigate interindividual variability, enhancing statistical power.

### 4.7 Recommendations for future research

Future research should explore force steadiness at various force levels and during isometric and dynamic contractions in people with pain, to understand changes in force control across different conditions. Both the CoV and the SD of force should be used for assessing force steadiness since they offer unique insights into force variability. Additionally, calculating SD in Newtons based on a submaximal percentage MVC can be problematic due to the non‐linear relationship between force output and MVC percentage, especially when considering inter‐individual differences in muscle strength. Thus, studies should express SD as a percentage or ensure both SD and the target are on the same scale.

Moreover, although most studies use the term force steadiness and report results in Newtons (for the SD), the measurements actually pertain to joint moments rather than muscle forces. Often, force transducers are attached at positions defined relative to anatomical landmarks, which can create differences in moment arms between individuals. These variations in moment arms can influence the calculated SD, as the lever arm length directly affects the torque produced by a given force. However, this variability should not affect the CoV, as it normalizes the SD by the mean force, thereby reducing the impact of individual differences in moment arm lengths. It is relevant to acknowledge this issue as it introduces an additional source of variability that future studies need to account for when measuring and interpreting force steadiness.

Lastly, given the significant role of visual feedback in force modulation (Limonta et al., 2015), future research should also investigate the underexplored influence of relying solely on proprioceptive input, in the absence of visual feedback, on force steadiness in individuals with musculoskeletal pain.

## 5 CONCLUSION

This review demonstrates that individuals with clinical pain exhibit decreased force steadiness, quantified by both the CoV and SD of force. It also reveals that force steadiness, quantified as CoV, is reduced in individuals subjected to experimentally induced pain, primarily induced through hypertonic saline injections. However, the results of the experimental pain studies should be interpreted with some caution. Future studies should explore the relationship between enhancements in force steadiness and improvements in patients' symptoms and functional performance.

## AUTHOR CONTRIBUTIONS

This review was conceived by Michail Arvanitidis, Deborah Falla and Eduardo Martinez‐Valdes; searches, study selection, data extraction and data handling were conducted by Michail Arvanitidis and Andy Sanderson. Michail Arvanitidis had a primary role in preparing the manuscript which was edited by Deborah Falla, Andy Sanderson and Eduardo Martinez‐Valdes. All authors have approved the final version of the manuscript and agree to be accountable for all aspects of the work.

## FUNDING INFORMATION

This research did not receive any specific grant from funding agencies in the public, commercial or not‐for‐profit sectors.

## CONFLICT OF INTEREST STATEMENT

The authors have no conflicts of interest to declare.

## Supporting information


Data S1.

Data S2.

Data S3.

Data S4.

## Data Availability

The template data collection forms, data extracted from included studies, data used for all analyses, analytic code and any other materials used in the review are available upon reasonable request. Interested researchers can contact the lead author, Michail Arvanitidis, for access to these materials.
