# Supplementary material for: Does pain influence control of muscle force? A systematic review and meta‐analysis
Source: Eur J Pain. 2024 Aug 23;29(2):e4716. doi: 10.1002/ejp.4716 (PMC11671343; doi:10.1002/ejp.4716)
Supplement: Supplementary file 2 — Data S2. [file EJP-29-0-s001.docx]

***Search Strategy (MEDLINE, Ovid Interface)***

1. Force.mp

2. Torque.mp

3. Moment.mp

4. 1-3 (OR)

5. Control.mp

6. Steadiness.mp

7. Variab*.mp

8. Modulat*.mp

9. 5-8 (OR)

10. Target.mp

11. Trajectory.mp

12. 10-11 (OR)

13. Coefficient of variation.mp

14. CoV.mp

15. Standard deviation.mp

16. SD.mp

17. Mean squar* error.mp

18. 13-17 (OR)

19. exp Pain/

20. exp Solutions/

21. Pain.mp

22. Acute Pain.mp

23. Chronic Pain.mp

24. Nociceptive pain.mp

25. Musculoskeletal pain.mp

26. Nocicept*.mp

27. Musculoskeletal disease*.mp

28. Hypertonic solution*.mp

29. Isotonic solution*.mp

30. Injections, Intramuscular.mp

31. Injections, Intra-Articular.mp

32. Electric stimulation.mp

33. 19-32 (OR)

34. **4 ADJ5 9**

35. **4 ADJ5 12**

36. **4 ADJ5 18**

37. **34 OR 35 OR 36 AND 33**

38. **Limit 37 to Humans**

***Search Strategy (EMBASE, Ovid Interface)***

1. Force.mp

2. Torque.mp

3. Moment.mp

4. 1-3 (OR)

5. Control.mp

6. Steadiness.mp

7. Variab*.mp

8. Modulat*.mp

9. 5-8 (OR)

10. Target.mp

11. Trajectory.mp

12. 10-11 (OR)

13. Coefficient of variation.mp

14. CoV.mp

15. Standard deviation.mp

16. SD.mp

17. Mean squar* error.mp

18. 13-17 (OR)

19. exp injection pain/

20. exp injection site pain/

21. exp musculoskeletal pain/

22. exp pain/

23. exp experimental muscle pain/

24. exp experimental pain/

25. exp nociceptive pain/

26. exp isotonic solution/

27. exp hypertonic solution/

28. Pain.mp

29. Acute Pain.mp

30. Chronic Pain.mp

31. Nociceptive pain.mp

32. Musculoskeletal pain.mp

33. Nocicept*.mp

34. Musculoskeletal disease*.mp

35. Hypertonic solution*.mp

36. Isotonic solution*.mp

37. Electric stimulation.mp

38. Intra-articular injection

39. Intramuscular injection

40. Intraarticular injection

41. Intra-muscular injection

42. 19-41 (OR)

43. **4 ADJ5 9**

44. **4 ADJ5 12**

45. **4 ADJ5 18**

46. **43 OR 44 OR 45 AND 42**

47. **Limit 46 to Humans**

***Search Strategy (PubMed)***

1. "Force control"

2. "Control of force"

3. "Force steadiness"

4. "Torque steadiness"

5. "Force variability"

6. "Torque variability"

7. "Force target"

8. "Torque target"

9. "Coefficient of Variation"

10. "Force SD"

11. "Torque SD"

12. 1-11 (OR)

13. Pain

14. Hypertonic solution*

15. Isotonic solution*

16. Nocicept*

17. Musculoskeletal disease*

18. "Musculoskeletal pain"[MeSH Terms]

19. 13-18 (OR)

20. **12 AND 19**

21. **Limit 20 to Humans**

***Search Strategy (CINAHL Plus, EBSCO Interface)***

S1 (force) n5 (control or steadiness or variab* or modulat*)

S2 (torque) n5 (control or steadiness or variab* or modulat*)

S3 (moment) n5 (control or steadiness or variab* or modulat*)

S4 (force) n5 (target or trajectory)

S5 (torque) n5 (target or trajectory)

S6 (moment) n5 (target or trajectory)

S7 (force) n5 (Coefficient of variation OR CoV OR Standard deviation OR SD OR Mean squar* error)

S8 (torque) n5 (Coefficient of variation OR CoV OR Standard deviation OR SD OR Mean squar* error)

S9 (moment) n5 (Coefficient of variation OR CoV OR Standard deviation OR SD OR Mean squar* error)

S10 (MH "Pain+" OR

(OR) MH "Nociceptive Pain+" OR

MH "Injections, Intramuscular+" OR

MM "Injections, Intraarticular" OR

MH "Hypertonic Solutions+" OR

MH "Isotonic Solutions+")

S11 (Pain OR

(OR) Acute Pain OR

Chronic Pain OR

Nociceptive pain OR

Musculoskeletal pain OR

Nocicept* OR

Musculoskeletal disease* OR

Hypertonic solution* OR

Isotonic solution* OR

Electric stimulation OR

Injections, Intramuscular OR

Injections, Intraarticular)

S12 **S1 OR S2 OR S3 OR S4 OR S5 OR S6 OR S7 OR S8 OR S9**

S13 **S10 OR S11**

S14 **S12 AND S13**

S15 **Limit S14 to Human**

***Search Strategy (Web of Science, Clarivate Analytics)***

1. TS=(force near/5  (control or steadiness or variab* or modulat*))

2. TS=(torque near/5  (control or steadiness or variab* or modulat*))

3. TS=(moment near/5  (control or steadiness or variab* or modulat*))

4. TS=(force near/5  (target or trajectory))

5. TS=(torque near/5  (target or trajectory))

6. TS=(moment near/5  (target or trajectory))

7. TS=(force near/5  ("Coefficient of variation" OR CoV OR "Standard deviation" OR SD OR "Mean squar* error"))

8. TS=(torque near/5  ("Coefficient of variation" OR CoV OR "Standard deviation" OR SD OR "Mean squar* error"))

9. TS=(moment near/5  ("Coefficient of variation" OR CoV OR "Standard deviation" OR SD OR "Mean squar* error"))

10. 1-9 (OR)

11. TS=(Pain OR

(OR) Acute Pain OR

Chronic Pain OR

Nociceptive Pain OR

Musculoskeletal pain OR

Nocicept* OR

Musculoskeletal disease* OR

Hypertonic Solution* OR

Isotonic Solution* OR

Electric stimulation OR

Intra-articular injection OR

Intramuscular injection OR

Intraarticular injection OR

Intra-muscular injection OR

Experimental pain)

12. **10 AND 11**
