# Supplementary material for: Does pain influence control of muscle force? A systematic review and meta‐analysis
Source: Eur J Pain. 2024 Aug 23;29(2):e4716. doi: 10.1002/ejp.4716 (PMC11671343; doi:10.1002/ejp.4716)
Supplement: Supplementary file 3 — Data S3. [file EJP-29-0-s003.docx]

***Changes for studies with clinical pain***

**Newcastle Ottawa Quality Assessment Scale**

**Cross-Sectional Studies**

*Adapted from Newcastle Ottawa Quality Assessment Scale for Case-Control Studies based on elements from the STROBE guidelines and the Newcastle Ottowa Scale manual (Wells, Accessed 2018, von Elm et al., 2008)*

Note: A study can be awarded a maximum of one star for each numbered item within the Selection and Exposure categories. A maximum of two stars can be given for Comparability.

**Selection**

1) Is the case definition adequate?

a) Yes, with independent validation 🟑

b) Yes, e.g. record linkage or based on self-reports

c) No description

2) Representativeness of the cases

a) Consecutive or obviously representative cases 🟑

b) Generally representative cases 🟑

c) Potential for selection biases or not stated

3) Selection of Controls

a) Community Controls 🟑

b) Alternative Control recruitment

c) No Description

4) Definition of Controls

a) No history of disease 🟑

b) No mention of history of outcome

**Comparability**

1) Comparability of cases and controls on the basis of the design or analysis

a) Study controls for two or more elements of the Population Demographics 🟑🟑

b) Study controls for any one element of the population demographics 🟑

c) Study does not control for any factor

**Exposure**

1) Description of Force/Torque steadiness Exposure

a) Force/Torque steadiness evaluation, recording equipment and processing are clearly described and standardised 🟑

b) Force/Torque steadiness evaluation, recording equipment and processing are sufficiently described and standardised 🟑

c) Force/Torque steadiness evaluation, recording equipment and processing are poorly described

2) Same Force/Torque steadiness Exposure for cases and controls

a) yes 🟑

b) no

3) Description of Task Exposure

a) Task was well described and implemented in a standardised well in both groups 🟑

b) Task was well described but not standardised

c) Task was poorly described and standardised

| **Question Number** | **Changes** | **Justification** |
| --- | --- | --- |
| **Section 1** | **Minor question wording changes** |  |
| 1 | No Changes made | This inquiry aims to determine the extent to which the symptoms or absence of symptoms in the Clinical-Experimental Pain/Con groups have been confirmed. |
| 2 | Addition of a second category | To address this issue, an extra classification has been introduced to acknowledge cases where the sample meets the eligibility criteria and participates but doesn't completely reflect the population under study. For instance, in the context of musculoskeletal pain (clinical or experimental), individuals with relatively low average pain levels (e.g., rated 2/10 on the PNRS) are included. Although these individuals may satisfy the inclusion criteria and exhibit no sampling bias, they do not fully represent the clinical population characterised by persistent high levels of pain and disability. |
| 3 | Modification of second category | The original wording assumed recruitment of cases exclusively from hospitals or through structured processes. The objective was to determine if the only distinction between cases and controls was the presence of case symptoms, while both originated from the same community. The second category aimed to identify whether controls were individuals from the hospital without the case, potentially representing a different background or community.  The revised phrasing accommodates the likelihood of cases not being recruited solely from a hospital, eliminating the option of hospital controls. Instead, the new classification considers whether cases and controls were sourced from the same population or if different sampling methods were employed for controls, potentially introducing selection bias. |
| 4 | Rewording of second category | This question examines whether bias may arise from including individuals who have previously experienced the case symptoms and whether such individuals should be excluded. The original wording of the question focused on the "source" of the case symptom, which is not relevant to the studies under review. The revised phrasing specifically addresses whether controls should have no prior history of the symptoms, ensuring the essence of the question is preserved. |
| **Section 2** | **Minor Alterations** |  |
| 5 | A third category has been included, and specific factors have been included. | The original question evaluates the extent to which cases and controls can be effectively compared and whether matching was incorporated during the design phase. The design allowed individual reviewers to choose the most relevant factors for their specific review. In this case, population demographics were selected as the factor to control for, considering that anthropomorphic features could potentially influence the primary outcome, force/torque steadiness.  As per the coding manual, if the specified criteria are not met or if group comparability occurs by chance after the fact, no stars should be assigned for this section. However, the original format did not provide an option for addressing this scenario. In this modification, an additional category has been included to ensure that this question is addressed and intentionally not assigned any stars, avoiding accidental omissions due to oversight. |
| **Section 3** | **Major Changes** | **Questions in this section have required the most modifications as the original section sought to ascertain if the cases and controls were exposed to the variable of interest. In studies included here, the variable of interest is the force/torque steadiness and by participating in the experiment, it is known that cases and controls are exposed.** |
| 6 | This question has been specifically rewritten for the force/torque steadiness exposure. | The original question aimed to evaluate the potential introduction of bias through the inadvertent inclusion of individuals as cases who have not been exposed to the variable of interest. This consideration is particularly important when tracking the progression of individuals with conditions like vertebral fractures over time, as the process of confirming or disproving the occurrence of a fracture can introduce bias. However, in this systematic review, there are two exposures to be considered: one to musculoskeletal pain and another to force/torque steadiness assessment and a specific task. The exposure to musculoskeletal pain cannot be externally verified due to its nature as an "invisible disability"; thus, all included studies would be categorised as "self-report" (as already considered in Q1).  The essence of this question lies in establishing the causality of results based on an individual's exposure to the variable of interest. In the context of assessing force steadiness, it is equally important to examine how the principles of force steadiness assessment are consistently applied across groups, including the choice of the dynamometer, task, position, and other relevant factors. Failure to standardise these aspects could introduce bias, making it challenging to determine if the results are attributable to an individual being a case or control or if the assessment of force steadiness was conducted accurately. |
| 7 | Slight change in the question wording | This question inquired whether the response to Q6 was consistent between cases and controls. Due to the modification of Q6, the title of this question has been slightly adjusted to align with this change. |
| 8 | This question has been specifically rewritten for the description of the force/torque steadiness exposure. | The original question assessed if reporting bias had been introduced through different numbers of participants in each group not responding to follow-up/the variable of interest. This question is not appropriate for the cross-sectional basis of this cohort as there is no follow-up period, and the outcome of interest is force/torque steadiness which, as a passive measurement technique, all participants will respond to.  The revised question evaluates whether the force/torque steadiness task was implemented consistently for all participants by determining if bias might have been introduced through non-standardized task instructions, ensuring uniformity in the assessment of force/torque steadiness across groups. |

**Thresholds were used to convert the Newcastle-Ottawa scales to AHRQ standards (good, fair, and poor):**

**Good quality:** 3 or 4 stars in selection domain AND 1 or 2 stars in comparability domain AND 2 or 3 stars in outcome/exposure domain.

**Fair quality:** 2 stars in selection domain AND 1 or 2 stars in comparability domain AND 2 or 3 stars in outcome/exposure domain.

**Poor quality:** 0 or 1 star in selection domain OR 0 stars in comparability domain OR 0 or 1 stars in outcome/exposure domain.

**Accessed Resources**

VON ELM, E., ALTMAN, D. G., EGGER, M., POCOCK, S. J., GOTZSCHE, P. C. & VANDENBROUCKE, J. P. 2008. The Strengthening the Reporting of Observational Studies in Epidemiology (STROBE) statement: guidelines for reporting observational studies. *J Clin Epidemiol,* 61**,** 344-9.

WELLS, G. A., SHEA, B., O'CONNELL, D., PETERSON, J., WELCH, V., LOSOS, M., TUGWELL, P. Accessed 2018. *The Newcastle-Ottawa Scale (NOS) for assessing the quality of nonrandomised studies in meta-analyses* [Online]. <http://www.ohri.ca/programs/clinical_epidemiology/oxford.asp>: Ottawa Hospital Research Institute. [Accessed 11/12/18 2018].

***Changes for studies with experimental pain***

**Newcastle Ottawa Quality Assessment Scale**

**Cohort Studies**

*Adapted from Newcastle Ottawa Quality Assessment Scale for Case-Control and Cohort Studies based on elements from the STROBE guidelines and the Newcastle Ottowa Scale manual (Wells, Accessed 2018, von Elm et al., 2008)*

Note: A study can be awarded a maximum of one star for each numbered item within the Selection and Exposure categories. A maximum of two stars can be given for Comparability.

**Selection**

1) Selection of Participants

a) Community Participants 🟑

b) Alternative Participant recruitment

c) No Description

2) Definition of Participants

a) No history of disease 🟑

b) No mention of history of outcome

3) Ascertainment of pain exposure

a) Pain was assessed, reported and it was >2/10 (NRS) 🟑

b) Minimal pain <2/10 (NRS) or no specific information on pain intensity and location

4) Demonstration that pain was not present at baseline

a) Yes 🟑

b) No

**Comparability**

1) Comparability Criteria in Within-Subject Design (Pain and No-Pain Conditions):

a) Randomisation was used for pain/no pain and order of testing 🟑🟑

b) Partial randomisation (one of the two) 🟑

c) No randomisation procedures

**Exposure**

1) Description of Force/Torque steadiness Exposure

a) Force/Torque steadiness evaluation, recording equipment and processing are clearly described and standardised 🟑

b) Force/Torque steadiness evaluation, recording equipment and processing are sufficiently described and standardised 🟑

c) Force/Torque steadiness evaluation, recording equipment and processing are poorly described

2) Same Force/Torque steadiness Exposure for controls during pain and no-pain conditions

a) yes 🟑

b) no

3) Description of Task Exposure

a) Task was well described and implemented in a standardised well in both conditions🟑

b) Task was well described but not standardised

c) Task was poorly described and standardised

| **Question Number** | **Changes** | **Justification** |
| --- | --- | --- |
| **Section 1** | **Major changes** |  |
| 1 | The original question 1 was removed and replaced by question number 3 (*case-control* document) | Considering that there are no cases/clinical pain group this question was completely removed for all the studies that used an experimental pain model and had a within-participant comparison. Question number 3 took its place, and the following changes were made to that question too:  The original wording in the document for case-control studies assumed recruitment of cases exclusively from hospitals or through structured processes. The objective was to determine if the only distinction between cases and controls was the presence of case symptoms, while both originated from the same community. The second category aimed to identify whether controls were individuals from the hospital without the case, potentially representing a different background or community.  The revised phrasing accommodates the likelihood of cases not being recruited solely from a hospital, eliminating the option of hospital controls. Instead, the new classification considers whether participants were sourced from the community or if different sampling methods were employed, potentially introducing selection bias. |
| 2 | The original question 2 was removed and replaced by question number 4 (*case-control* document) | Considering that there are no cases/clinical pain group this section was removed entirely for all the studies that used an experimental pain model and had a within-participant comparison. Question number 4 took its place, and the following changes were made to that question too:  This question examines whether bias may arise from including individuals who have previously experienced the case symptoms and whether such individuals should be excluded. The original wording of the question in the case-control document focused on the "source" of the case symptom, which is not relevant to the studies under review. The revised phrasing specifically addresses whether participants should have no prior history of the symptoms, ensuring the essence of the question is preserved. |
| 3 | Used from the *cohort-studies* document and heavily modified | The title of this section was revised to explicitly reference exposure to painful stimuli. This modification was necessary to accurately reflect the nature of the studies discussed. To streamline the focus, the subcategories have been condensed into two distinct groups. The first group encompasses studies where participants experienced pain exceeding 2/10 on the Numeric Rating Scale (NRS), ensuring that the stimuli were sufficiently intense to replicate a genuine pain scenario. The second group includes studies where pain was either below this threshold or where specific details about the pain's intensity and location were not provided. |
| 4 | Used from the *cohort-studies* document and slightly modified | The title of this section has been updated to clearly indicate that the primary focus is on pain as an outcome. This subcategory specifically examines the methodological rigor of the studies, particularly in terms of testing sequence and overall design. The objective is to ascertain whether appropriate controls were in place to guarantee that participants were not experiencing pain at the start of the study, and this was demonstrated by a measurement of pain level in participants at the point of injection. This assessment is crucial to ensure the validity of the results, as it helps to determine whether any observed pain is a direct consequence of the experimental conditions rather than a pre-existing condition. |
| **Section 2** | **Minor Changes** |  |
| 5 | The wording has changed, and two stars are only given for the first answer | This section has been updated to control for confounding factors and biases, specifically in the context of within-subject designs focusing on pain and no-pain conditions. This revision categorises studies based on the use of randomisation (pain model, order of testing), emphasising its role in enhancing methodological rigor. The first category includes studies that implement full randomisation for both pain/no pain conditions and the order of testing (two stars). The second category encompasses studies that apply partial randomisation, either to the pain/no pain conditions or the order of testing. (one star). The final category is reserved for studies that do not employ any randomisation procedures (no stars). These changes aim to provide a more detailed and accurate assessment of each study's approach to managing potential biases and confounding factors, thereby ensuring comparability between painful and non-painful statuses in pain research. |
| **Section 3** | **Major Changes** | **Questions in this section have required the most modifications as the original section sought to ascertain if the cases and controls were exposed to the variable of interest. In studies included here, the variable of interest is the force/torque steadiness and by participating in the experiment, it is known that cases and controls are exposed.** |
| 6 | This question has been specifically rewritten for the force/torque steadiness exposure. | The original question aimed to evaluate the potential introduction of bias through the inadvertent inclusion of individuals as cases who have not been exposed to the variable of interest. This consideration is particularly important when tracking the progression of individuals with conditions like vertebral fractures over time, as the process of confirming or disproving the occurrence of a fracture can introduce bias. However, in this systematic review, there are two exposures to be considered: one to musculoskeletal pain and another to force/torque steadiness assessment and a specific task. The exposure to musculoskeletal pain cannot be externally verified due to its nature as an "invisible disability"; thus, all included studies would be categorised as "self-report" (as already considered in Q1).  The essence of this question lies in establishing the causality of results based on an individual's exposure to the variable of interest. In the context of assessing force steadiness, it is equally important to examine how the principles of force steadiness assessment are consistently applied across conditions (pain, no-pain), including the choice of the dynamometer, task, position, and other relevant factors. Failure to standardise these aspects could introduce bias, making it challenging to determine if the results are attributable to an individual being a case or control or if the assessment of force steadiness was conducted accurately. |
| 7 | Slight change in the question wording | This question inquired whether the response to Q6 was consistent between the two conditions (pain, no-pain). Due to the modification of Q6, the title of this question has been slightly adjusted to align with this change. |
| 8 | This question has been specifically rewritten for the description of the force/torque steadiness exposure. | The original question assessed if reporting bias had been introduced through different numbers of participants in each group not responding to follow-up/the variable of interest. This question is not appropriate for the cross-sectional basis of this cohort as there is no follow-up period, and the outcome of interest is force/torque steadiness which, as a passive measurement technique, all participants will respond to.  The revised question evaluates whether the force/torque steadiness task was implemented consistently for all participants by determining if bias might have been introduced through non-standardized task instructions, ensuring uniformity in the assessment of force/torque steadiness across conditions (pain, no-pain). |

**Thresholds were used to convert the Newcastle-Ottawa scales to AHRQ standards (good, fair, and poor):**

**Good quality:** 3 or 4 stars in selection domain AND 1 or 2 stars in comparability domain AND 2 or 3 stars in outcome/exposure domain.

**Fair quality:** 2 stars in selection domain AND 1 or 2 stars in comparability domain AND 2 or 3 stars in outcome/exposure domain.

**Poor quality:** 0 or 1 star in selection domain OR 0 stars in comparability domain OR 0 or 1 stars in outcome/exposure domain.

**Accessed Resources**

VON ELM, E., ALTMAN, D. G., EGGER, M., POCOCK, S. J., GOTZSCHE, P. C. & VANDENBROUCKE, J. P. 2008. The Strengthening the Reporting of Observational Studies in Epidemiology (STROBE) statement: guidelines for reporting observational studies. *J Clin Epidemiol,* 61**,** 344-9.

WELLS, G. A., SHEA, B., O'CONNELL, D., PETERSON, J., WELCH, V., LOSOS, M., TUGWELL, P. Accessed 2018. *The Newcastle-Ottawa Scale (NOS) for assessing the quality of nonrandomised studies in meta-analyses* [Online]. <http://www.ohri.ca/programs/clinical_epidemiology/oxford.asp>: Ottawa Hospital Research Institute. [Accessed 11/12/18 2018].
