# Supplementary material for: Does pain influence control of muscle force? A systematic review and meta‐analysis
Source: Eur J Pain. 2024 Aug 23;29(2):e4716. doi: 10.1002/ejp.4716 (PMC11671343; doi:10.1002/ejp.4716)
Supplement: Supplementary file 4 — Data S4. [file EJP-29-0-s004.docx]

**Sensitivity & Influence Analyses for Clinical Pain Studies**

***Influence analysis for CoV-Clinical Pain Studies***

***Fig. A:*** The figure presents an influence analysis for the meta-analytic model, illustrating various diagnostic measures to identify influential studies. The plots include externally studentized residuals, DFFITS values, Cook's distances, covariance ratios, estimates of heterogeneity (τ² & Q-statistic) when each study is removed, hat values, weights and DFBETAS. On the y-axis are the values of each measure, and on the x-axis are the study numbers, with the first author of each study listed in the adjacent table. The blue line in each plot represents the mean of each parameter across all studies. Studies that cause significant changes in these diagnostics when excluded are considered influential, indicating their substantial impact on the model's fit and the overall results. If there is an influential case, this is depicted with a red asterisk next to the author's name in the table.

***Influence analysis for SD-Clinical Pain Studies***

***Fig. B:*** The figure presents an influence analysis for the meta-analytic model, illustrating various diagnostic measures to identify influential studies. The plots include externally studentized residuals, DFFITS values, Cook's distances, covariance ratios, estimates of heterogeneity (τ² & Q-statistic) when each study is removed, hat values, weights and DFBETAS. On the y-axis are the values of each measure, and on the x-axis are the study numbers, with the first author of each study listed in the adjacent table. The blue line in each plot represents the mean of each parameter across all studies. Studies that cause significant changes in these diagnostics when excluded are considered influential, indicating their substantial impact on the model's fit and the overall results. If there is an influential case, this is depicted with a red asterisk next to the author's name in the table.

***Sensitivity Analyses for CoV – Leaving One Study Out***

***Table A.*** The table presents the results of a sensitivity analysis conducted by leaving one study out at a time to evaluate the influence on the overall combined effect size for **Force/Torque CoV** on clinical pain studies. The analysis aims to determine how each individual study impacts the robustness and consistency of the overall findings.

| Excluded study.  Author, year | Combined effect size (95% CI) | p-value | I^2^(%; 95% CI) | Q | Tau^2^ | df |
| --- | --- | --- | --- | --- | --- | --- |
| Arvanitidis 2022 | 0.80 [0.28, 1.33] | ***0.006*** | 89 [83, 92] | 124.06 | 0.60 | 14 |
| Arvanitidis 2023 (Trunk Ext & Flex) | 0.79 [0.26, 1.33] | ***0.007*** | 89 [84, 93] | 122.86 | 0.61 | 13 |
| Bandholm 2006 | 0.78 [0.26, 1.30] | ***0.006*** | 89 [83, 92] | 123.47 | 0.59 | 14 |
| Camargo 2009 | 0.86 [0.35, 1.37] | ***0.003*** | 88 [82, 92] | 119.19 | 0.56 | 14 |
| Falla 2010 | 0.83 [0.31, 1.35] | ***0.004*** | 89 [83, 92] | 123.61 | 0.59 | 14 |
| Ferreira 2021 (Hip & Knee) | 0.67 [0.21, 1.13] | ***0.007*** | 84 [74, 90] | 80.29 | 0.42 | 13 |
| Magni, 2021 | 0.68 [0.22, 1.15] | ***0.007*** | 86 [78, 91] | 98.43 | 0.45 | 14 |
| Miura, 2014 | 0.83 [0.31, 1.35] | ***0.004*** | 89 [83, 92] | 123.26 | 0.59 | 14 |
| Muceli, 2011 | 0.75 [0.23, 1.27] | ***0.008*** | 88 [83, 92] | 120.38 | 0.58 | 14 |
| Overbeek, 2020 (Abd & Add) | 0.93 [0.48, 1.39] | ***<0.001*** | 79 [65, 87] | 61.84 | 0.41 | 13 |
| Testa, 2017 | 0.86 [0.35, 1.37] | ***0.003*** | 88 [83, 92] | 120.94 | 0.55 | 14 |
| Wang, 2018 | 0.80 [0.27, 1.33] | ***0.006*** | 89 [83, 92] | 124.01 | 0.61 | 14 |
| Wang, 2020 | 0.79 [0.27, 1.32] | ***0.006*** | 89 [83, 92] | 123.90 | 0.60 | 14 |

**Significant results are highlighted by bolding the p-values.

***Sensitivity Analyses for SD – Leaving One Study Out***

***Table B.*** The table presents the results of a sensitivity analysis conducted by leaving one study out at a time to evaluate the influence on the overall combined effect size for **Force/Torque SD** on clinical pain studies. The analysis aims to determine how each individual study impacts the robustness and consistency of the overall findings.

| Excluded study.  Author, year | Combined effect size (95% CI) | p-value | I^2^(%; 95% CI) | Q | Tau^2^ | df |
| --- | --- | --- | --- | --- | --- | --- |
| Arvanitidis, 2022 | 0.61 [0.07, 1.15] | ***0.030*** | 88 [81, 92] | 113.97 | 0.72 | 14 |
| Arvanitidis 2023 (Trunk Ext & Flex) | 0.56 [0.01, 1.11] | ***0.047*** | 88 [81, 92] | 107.23 | 0.73 | 13 |
| Bandholm, 2006 | 0.59 [0.06, 1.13] | ***0.032*** | 88 [81, 92] | 113.52 | 0.71 | 14 |
| Camargo, 2009 | 0.64 [0.10, 1.18] | ***0.023*** | 88 [81, 92] | 113.46 | 0.71 | 14 |
| Hortobagyi,2004 | 0.49 [0.02, 0.96] | ***0.043*** | 85 [77, 90] | 94.84 | 0.53 | 14 |
| Magni, 2021 | 0.48 [0.02, 0.95] | ***0.044*** | 82 [71, 89] | 77.30 | 0.50 | 14 |
| Mista, 2018 | 0.70 [0.20, 1.20] | ***0.010*** | 86 [79, 91] | 102.77 | 0.60 | 14 |
| Miura, 2014 | 0.64 [0.11, 1.18] | ***0.022*** | 88 [81, 92] | 113.46 | 0.70 | 14 |
| Overbeek, 2020 (Abd & Add) | 0.72 [0.22, 1.22] | ***0.008*** | 81 [70, 88] | 69.23 | 0.56 | 13 |
| Testa, 2015 | 0.66 [0.13, 1.18] | ***0.019*** | 88 [81, 92] | 112.74 | 0.69 | 14 |
| Testa, 2017 | 0.66 [0.13, 1.19] | ***0.018*** | 88 [81, 92] | 112.40 | 0.69 | 14 |
| Testa, 2018 | 0.57 [0.04, 1.10] | ***0.037*** | 87 [81, 92] | 111.50 | 0.69 | 14 |
| Wang, 2018 | 0.60 [0.06, 1.14] | ***0.031*** | 88 [81, 92] | 113.69 | 0.72 | 14 |
| Wang, 2020 | 0.60 [0.06, 1.14] | ***0.032*** | 88 [81, 92] | 113.58 | 0.72 | 14 |

**Significant results are highlighted by bolding the p-values.

***Sensitivity Analyses for CoV – Forest Plot – (excluding studies that did not provide visual force feedback)***

***Fig. C.*** This figure presents a forest plot of the sensitivity analysis conducted after removing Torque CoV data from the study by Testa et al., 2017, during the condition without visual feedback. This step was taken to ensure that the confounding factor of visual feedback does not influence the overall analysis. The meta-analysis examines the effect of clinical pain on force steadiness during different submaximal voluntary contractions. The mean ± SD of each outcome measure and sample size for each group are reported, alongside the standardized mean difference (SMD) and 95% confidence interval (95% CI) for each study. All heterogeneity measures and overall effect size tests are reported below the forest plot, with the prediction interval depicted as a red line within the graph. The forest plots are organized in ascending order of their effect sizes.


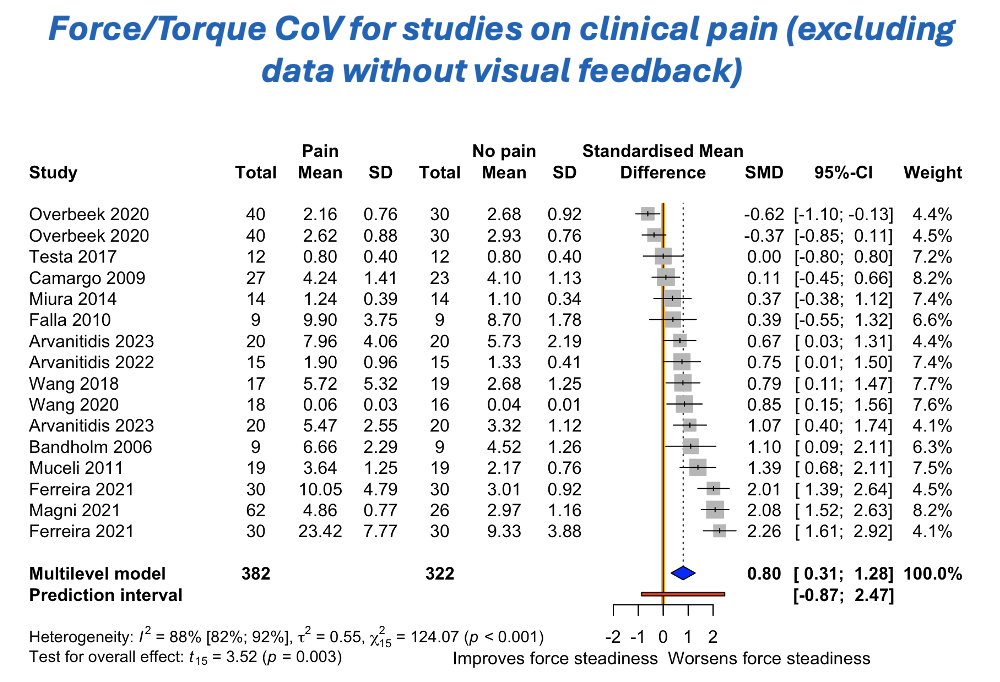


***Sensitivity Analyses for SD – Forest Plot – (excluding studies that did not provide visual force feedback)***

***Fig. D.*** This figure presents a forest plot of the sensitivity analysis conducted after removing Torque SD data from the study by Testa et al., 2017 and 2018, during the condition without visual feedback. This step was taken to ensure that the confounding factor of visual feedback does not influence the overall analysis. The meta-analysis examines the effect of clinical pain on force steadiness during different submaximal voluntary contractions. The mean ± SD of each outcome measure and sample size for each group are reported, alongside the standardized mean difference (SMD) and 95% confidence interval (95% CI) for each study. All heterogeneity measures and overall effect size tests are reported below the forest plot, with the prediction interval depicted as a red line within the graph. The forest plots are organized in ascending order of their effect sizes.

***Sensitivity Analyses for CoV – Forest Plot – Removing Studies of Poor Quality***

***Fig. E.*** This figure presents a forest plot of the sensitivity analysis conducted after removing studies of poor quality (presented in the table next to the forest plot). This step was taken to explore how removing these studies of high risk of bias will influence the overall analysis. The meta-analysis examines the effect of clinical pain on force steadiness during different submaximal voluntary contractions. The mean ± SD of each outcome measure and sample size for each group are reported, alongside the standardized mean difference (SMD) and 95% confidence interval (95% CI) for each study. All heterogeneity measures and overall effect size tests are reported below the forest plot, with the prediction interval depicted as a red line within the graph. The forest plots are organized in ascending order of their effect sizes.


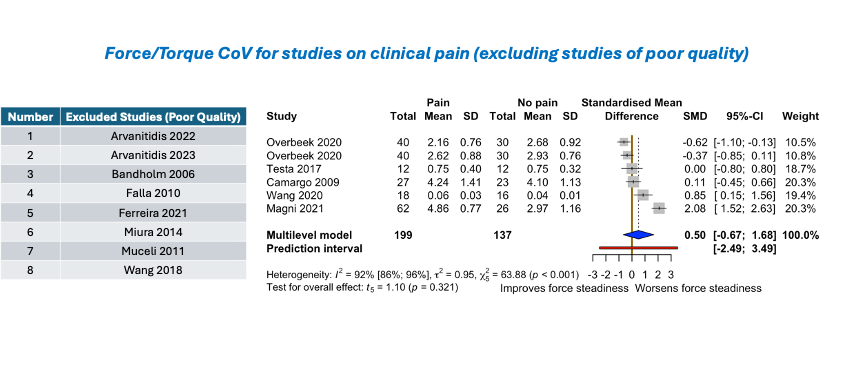


***Sensitivity Analyses for SD – Forest Plot – Removing Studies of Poor Quality***

***Fig. F.*** This figure presents a forest plot of the sensitivity analysis conducted after removing studies of poor quality (presented in the table next to the forest plot). This step was taken to explore how removing these studies of high risk of bias will influence the overall analysis. The meta-analysis examines the effect of clinical pain on force steadiness during different submaximal voluntary contractions. The mean ± SD of each outcome measure and sample size for each group are reported, alongside the standardized mean difference (SMD) and 95% confidence interval (95% CI) for each study. All heterogeneity measures and overall effect size tests are reported below the forest plot, with the prediction interval depicted as a red line within the graph. The forest plots are organized in ascending order of their effect sizes.


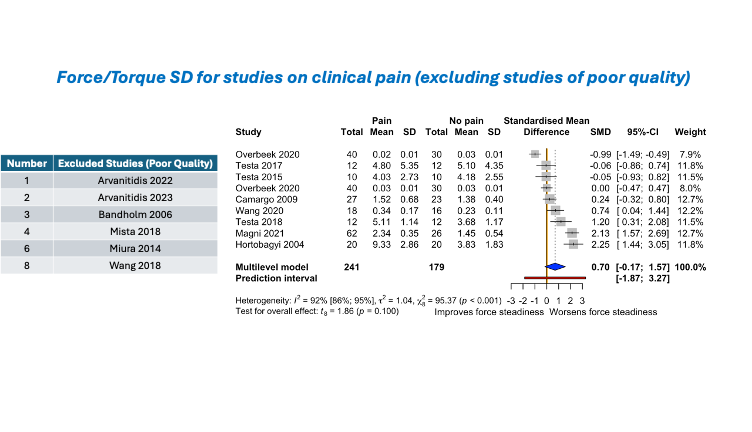


**Sensitivity & Influence Analyses for Experimental Pain Studies**

***Influence analysis for CoV-Experimental Pain Studies***

***Fig. G:*** The figure presents an influence analysis for the meta-analytic model, illustrating various diagnostic measures to identify influential studies. The plots include externally studentized residuals, DFFITS values, Cook's distances, covariance ratios, estimates of heterogeneity (τ² & Q-statistic) when each study is removed, hat values, weights and DFBETAS. On the y-axis are the values of each measure, and on the x-axis are the study numbers, with the first author of each study listed in the adjacent table. The blue line in each plot represents the mean of each parameter across all studies. Studies that cause significant changes in these diagnostics when excluded are considered influential, indicating their substantial impact on the model's fit and the overall results. If there is an influential case, this is depicted with a red asterisk next to the author's name in the table.


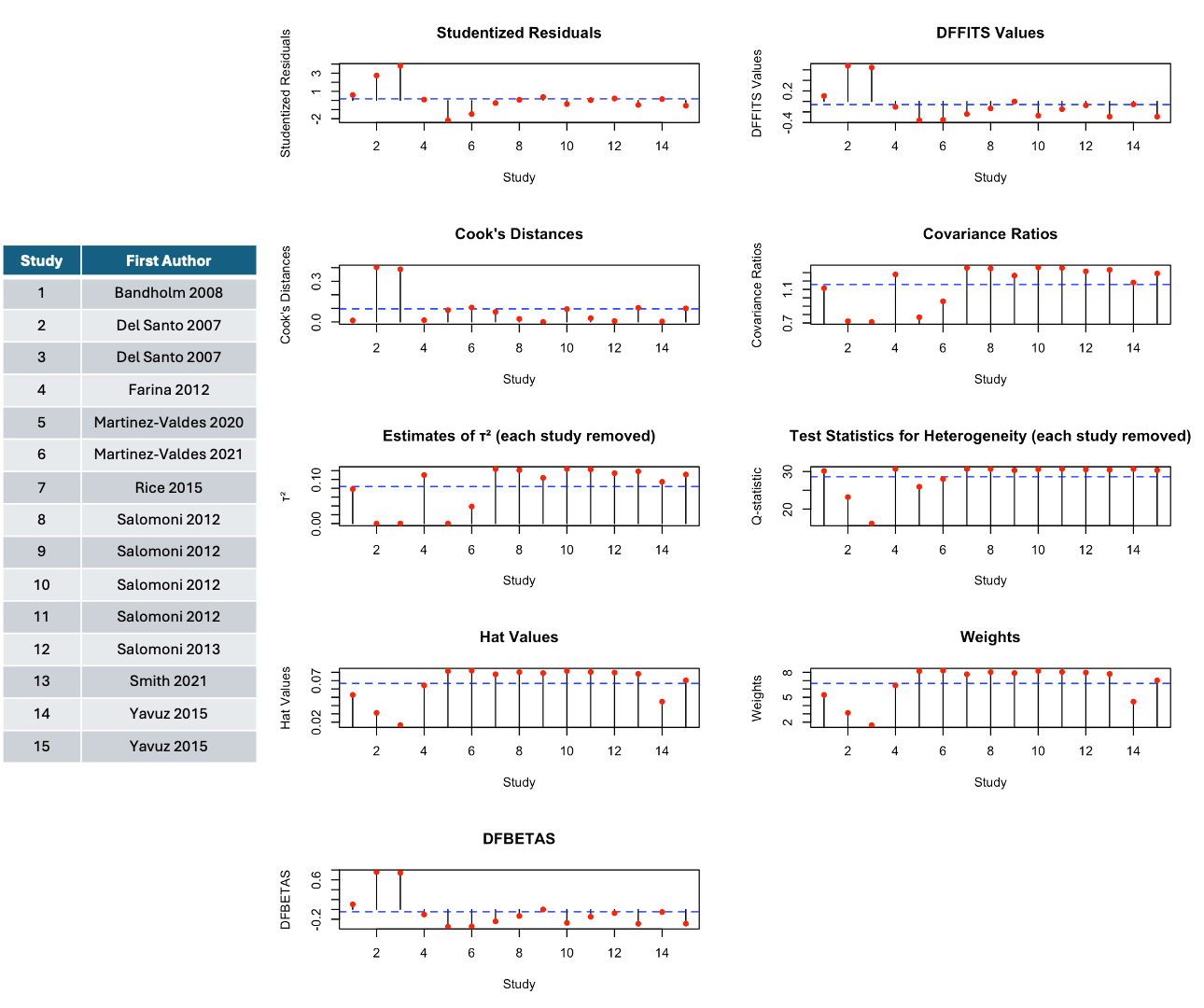


***Influence analysis for SD-Experimental Pain Studies***

***Fig. H:*** The figure presents an influence analysis for the meta-analytic model, illustrating various diagnostic measures to identify influential studies. The plots include externally studentized residuals, DFFITS values, Cook's distances, covariance ratios, estimates of heterogeneity (τ² & Q-statistic) when each study is removed, hat values, weights and DFBETAS. On the y-axis are the values of each measure, and on the x-axis are the study numbers, with the first author of each study listed in the adjacent table. The blue line in each plot represents the mean of each parameter across all studies. Studies that cause significant changes in these diagnostics when excluded are considered influential, indicating their substantial impact on the model's fit and the overall results. If there is an influential case, this is depicted with a red asterisk next to the author's name in the table.


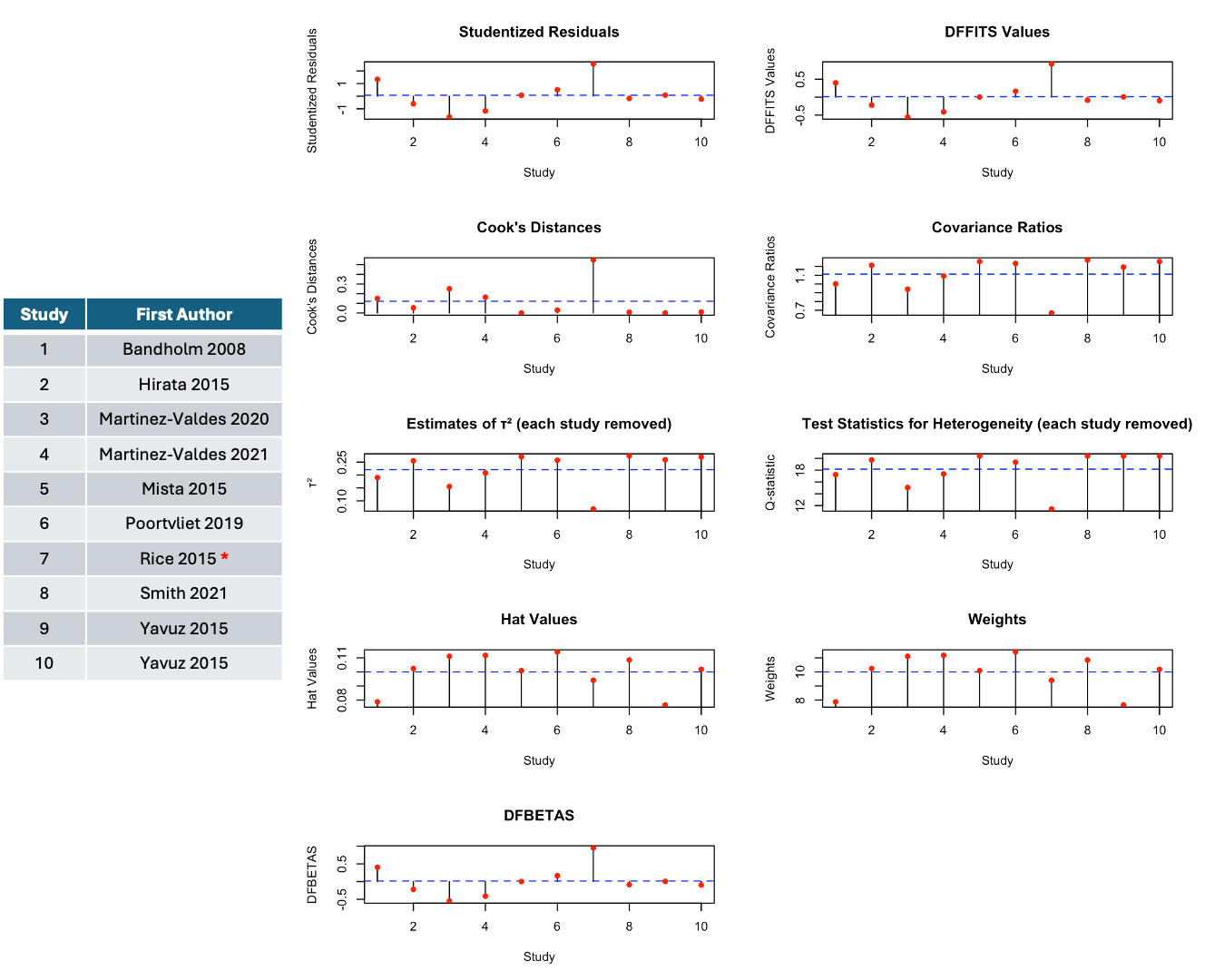


**Sensitivity Analyses for Experimental Pain Studies**

***Table C.*** The table presents the results of a sensitivity analysis conducted by leaving one study out at a time to evaluate the influence on the overall combined effect size for **Force/Torque CoV** on experimental pain studies. The analysis aims to determine how each individual study impacts the robustness and consistency of the overall findings.

| Excluded study.  Author, year | Combined effect size (95% CI) | p-value | I^2^(%; 95% CI) | Q | Tau^2^ | df |
| --- | --- | --- | --- | --- | --- | --- |
| Bandholm 2008 | 0.48 [-0.07, 1.03] | *0.082* | 57 [22, 76] | 30.12 | 0.44 | 13 |
| Del Santo 2007 (Elbow Flex & Fifth finger Abd) | 0.34 [0.07, 0.60] | ***0.017*** | 0 [0, 58] | 8.15 | <0.01 | 13 |
| Farina 2012 | 0.51 [-0.06, 1.07] | *0.074* | 58 [23, 77] | 30.66 | 0.47 | 13 |
| Martinez-Valdes 2020 | 0.59 [0.09, 1.09] | ***0.024*** | 50 [7, 73] | 25.92 | 0.33 | 13 |
| Martinez-Valdes 2021 | 0.58 [0.04, 1.11] | ***0.036*** | 54 [15, 75] | 28.00 | 0.40 | 13 |
| Rice 2015 | 0.53 [-0.04, 1.10] | *0.066* | 58 [23, 77] | 30.66 | 0.47 | 13 |
| Salomoni 2012 (All muscles) | 0.51 [-0.09, 1.12] | *0.087* | 67 [37, 82] | 30.07 | 0.50 | 10 |
| Salomoni 2013 | 0.50 [-0.07, 1.07] | *0.079* | 57 [23, 77] | 30.52 | 0.47 | 13 |
| Smith 2021 | 0.54 [-0.03, 1.10] | *0.060* | 57 [23, 76] | 30.45 | 0.46 | 13 |
| Yavuz 2015 (Ankle DF & Fifth Finger Abd) | 0.58 [-0.09, 1.25] | *0.085* | 63 [32, 80] | 29.98 | 0.59 | 13 |

**Significant results are highlighted by bolding the p-values.

***Table D.*** The table presents the results of a sensitivity analysis conducted by leaving one study out at a time to evaluate the influence on the overall combined effect size for **Force/Torque SD** on experimental pain studies. The analysis aims to determine how each individual study impacts the robustness and consistency of the overall findings.

| Excluded study.  Author, year | Combined effect size (95% CI) | p-value | I^2^(%; 95% CI) | Q | Tau^2^ | df |
| --- | --- | --- | --- | --- | --- | --- |
| Bandholm 2008 | 0.36 [-0.13, 0.85] | *0.131* | 54 [1, 78] | 17.23 | 0.22 | 8 |
| Hirata 2015 | 0.49 [-0.06, 1.04] | *0.072* | 59 [15, 81] | 19.73 | 0.29 | 8 |
| Martinez-Valdes 2020 | 0.55 [0.07, 1.03] | ***0.029*** | 47 [0, 75] | 15.06 | 0.18 | 8 |
| Martinez-Valdes 2021 | 0.53 [0.01, 1.05] | ***0.027*** | 54 [2, 78] | 17.36 | 0.24 | 8 |
| Mista 2015 | 0.44 [-0.12, 1.00] | *0.105* | 61 [19, 81] | 20.38 | 0.31 | 8 |
| Poortvliet 2019 | 0.41 [-0.15, 0.96] | *0.129* | 59 [14, 80] | 19.35 | 0.30 | 8 |
| Rice 2015 | 0.29 [-0.10, 0.68] | *0.128* | 30 [0, 68] | 11.42 | 0.08 | 8 |
| Smith 2021 | 0.46 [-0.10, 1.03] | *0.094* | 61 [19, 81] | 20.41 | 0.32 | 8 |
| Yavuz 2015 (Ankle DF & Fifth Finger Abd) | 0.46 [-0.13, 1.05] | *0.110* | 66 [27, 84] | 20.35 | 0.32 | 7 |

**Significant results are highlighted by bolding the p-values.

***Sensitivity Analyses for CoV – Forest Plot – (excluding studies that did not provide visual force feedback)***

***Fig. I.*** This figure presents a forest plot of the sensitivity analysis conducted after removing Torque CoV data from the study by Smith et al., 2021, during the condition without visual feedback. This step was taken to ensure that the confounding factor of visual feedback does not influence the overall analysis. The meta-analysis examines the effect of clinical pain on force steadiness during different submaximal voluntary contractions. The mean ± SD of each outcome measure and sample size for each group are reported, alongside the standardized mean difference (SMD) and 95% confidence interval (95% CI) for each study. All heterogeneity measures and overall effect size tests are reported below the forest plot, with the prediction interval depicted as a red line within the graph. The forest plots are organized in ascending order of their effect sizes.


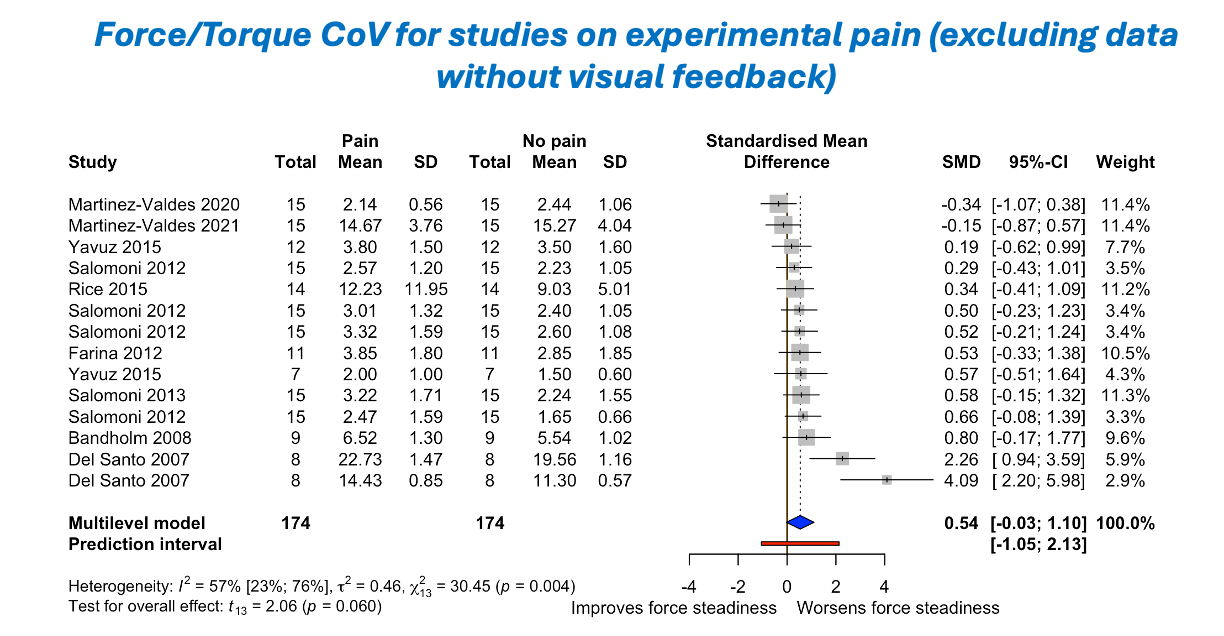


This analysis resulted in a change of the overall effect (i.e., there was no influence of experimental pain on force steadiness). However, this exclusion resulted in identifying a potential outlier in our analysis. Specifically, the data from Del Santo 2007 were identified as potential outliers after performing the influence analysis presented below:

***Additional Influence analysis for CoV-Experimental Pain Studies (excluding studies that did not provide visual force feedback)***

***Fig. J:*** The figure presents an influence analysis for the meta-analytic model after the exclusion of the study by Smith et al., 2021, which did not use visual feedback, illustrating various diagnostic measures to identify influential studies. The plots include externally studentized residuals, DFFITS values, Cook's distances, covariance ratios, estimates of heterogeneity (τ² & Q-statistic) when each study is removed, hat values, weights and DFBETAS. On the y-axis are the values of each measure, and on the x-axis are the study numbers, with the first author of each study listed in the adjacent table. The blue line in each plot represents the mean of each parameter across all studies. Studies that cause significant changes in these diagnostics when excluded are considered influential, indicating their substantial impact on the model's fit and the overall results. If there is an influential case, this is depicted with a red asterisk next to the author's name in the table.

***
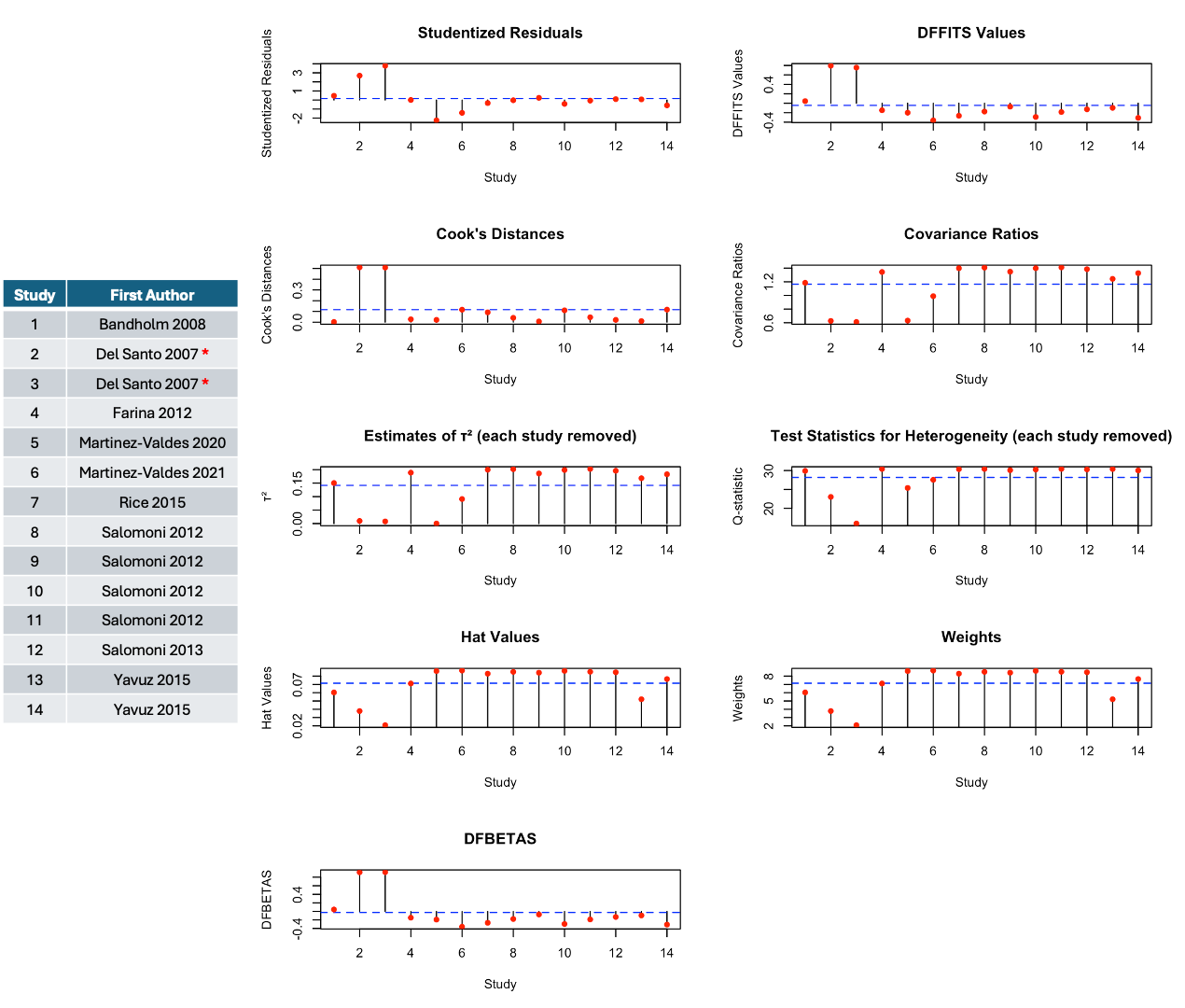
***

This analysis showed that after removing the study by Smith et al., 2021, the study by Del Santo et al., 2007 was detected as a potential outlier. Therefore, we excluded this study to further explore how the overall effect of our meta-analysis would be affected. This is presented in the forest plot below:

***Additional Sensitivity Analyses for CoV – Forest Plot – (excluding studies that did not provide visual force feedback)***

***Fig. K.*** This figure presents a forest plot of the sensitivity analysis conducted after removing Torque CoV data from the study by Smith et al., 2021, during the condition without visual feedback and a potential outlier study by Del Santo et al., 2007. This step was taken to ensure that the confounding factor of visual feedback and a potential outlier does not influence the overall analysis. The meta-analysis examines the effect of clinical pain on force steadiness during different submaximal voluntary contractions. The mean ± SD of each outcome measure and sample size for each group are reported, alongside the standardized mean difference (SMD) and 95% confidence interval (95% CI) for each study. All heterogeneity measures and overall effect size tests are reported below the forest plot, with the prediction interval depicted as a red line within the graph. The forest plots are organized in ascending order of their effect sizes.


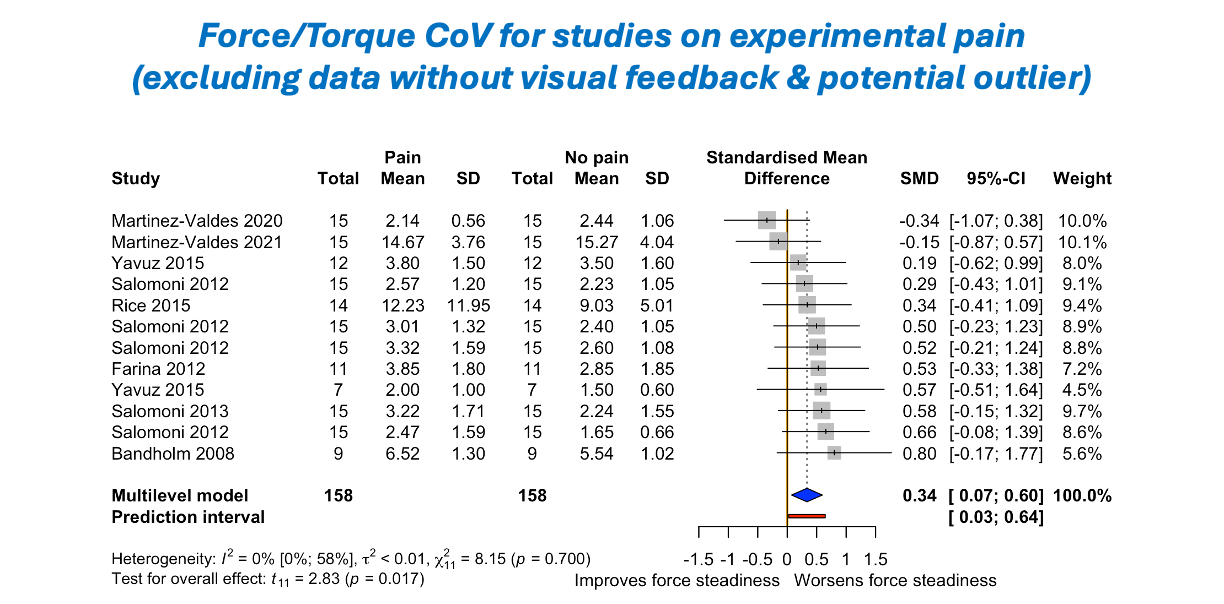


Removing the study that performed the tests without visual feedback and a potential outlier resulted in observing the same result of our main analysis (i.e., force steadiness being impaired in the presence of experimentally induced pain) and importantly led to a significant reduction in heterogeneity.

***Sensitivity Analyses for SD – (excluding studies that did not provide visual force feedback)***

***Fig. L.*** This figure presents a forest plot of the sensitivity analysis conducted after removing Torque SD data from the study by Smith et al., 2021, during the condition without visual feedback. This step was taken to ensure that the confounding factor of visual feedback does not influence the overall analysis. The meta-analysis examines the effect of clinical pain on force steadiness during different submaximal voluntary contractions. The mean ± SD of each outcome measure and sample size for each group are reported, alongside the standardized mean difference (SMD) and 95% confidence interval (95% CI) for each study. All heterogeneity measures and overall effect size tests are reported below the forest plot, with the prediction interval depicted as a red line within the graph. The forest plots are organized in ascending order of their effect sizes.


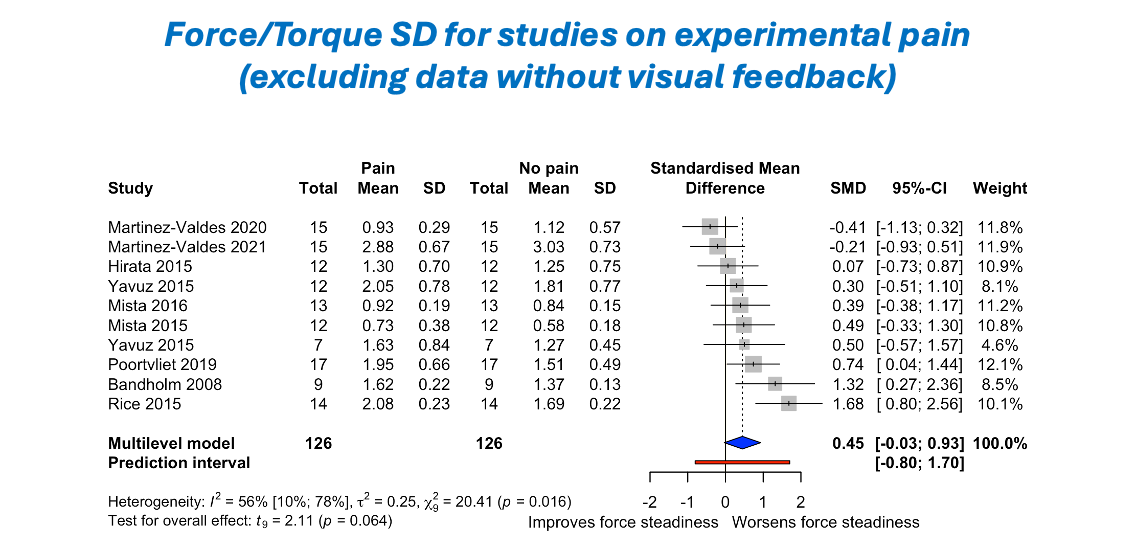


***Sensitivity Analyses for CoV – Forest Plot – Removing Studies of Poor Quality***

***Fig. M.*** This figure presents a forest plot of the sensitivity analysis conducted after removing studies of poor quality (presented in the table next to the forest plot). This step was taken to explore how removing these studies of high risk of bias will influence the overall analysis. The meta-analysis examines the effect of clinical pain on force steadiness during different submaximal voluntary contractions. The mean ± SD of each outcome measure and sample size for each group are reported, alongside the standardized mean difference (SMD) and 95% confidence interval (95% CI) for each study. All heterogeneity measures and overall effect size tests are reported below the forest plot, with the prediction interval depicted as a red line within the graph. The forest plots are organized in ascending order of their effect sizes.


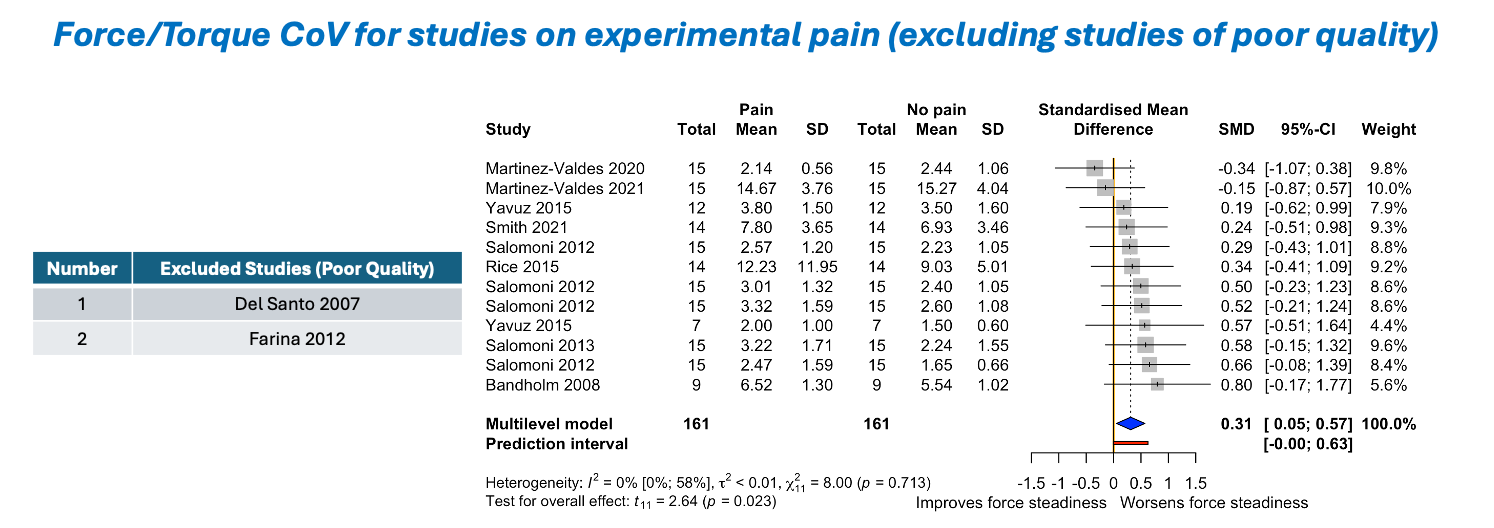


This analysis was not performed for the force/torque SD-based meta-analysis, as none of the studies were of poor quality.
